# Design of novel peptide inhibitors against the conserved bacterial transcription terminator, Rho

**DOI:** 10.1016/j.jbc.2021.100653

**Published:** 2021-05-15

**Authors:** Gairika Ghosh, Pankaj V. Sharma, Amit Kumar, Sriyans Jain, Ranjan Sen

**Affiliations:** 1 Laboratory of Transcription, Center for DNA Fingerprinting and Diagnostics, Uppal, Hyderabad, India; 2 Graduate Studies, Manipal Institute of Higher Education, Manipal, Karnataka, India

**Keywords:** Rho, Psu, peptides, transcription termination, bacteriophage, AMP, antimicrobial peptides, ATc, anhydrous tetracycline, BSA, bovine serum albumin, CTD, C-terminal domain, EC, elongation complex, ITC, isothermal calorimetry, KFC, Knowledge-based FADE and Contacts, NTD, N-terminal domain, Ni-NTA, nickel nitrilotriacetic acid, PBS, primary RNA-binding site, qRT-PCR, quantitative reverse transcription PCR, RB, roadblock, SBS, secondary RNA-binding site, T-buffer, transcription buffer

## Abstract

The transcription terminator Rho regulates many physiological processes in bacteria, such as antibiotic sensitivity, DNA repair, RNA remodeling, and so forth, and hence, is a potential antimicrobial target, which is unexplored. The bacteriophage P4 capsid protein, Psu, moonlights as a natural Rho antagonist. Here, we report the design of novel peptides based on the C-terminal region of Psu using phenotypic screening methods. The resultant 38-mer peptides, in addition to containing mutagenized Psu sequences, also contained plasmid sequences, fused to their C termini. Expression of these peptides inhibited the growth of *Escherichia coli* and specifically inhibited Rho-dependent termination *in vivo*. Peptides 16 and 33 exhibited the best Rho-inhibitory properties *in vivo.* Direct high-affinity binding of these two peptides to Rho also inhibited the latter's RNA-dependent ATPase and transcription termination functions *in vitro*. These two peptides remained functional even if eight to ten amino acids were deleted from their C termini. *In silico* modeling and genetic and biochemical evidence revealed that these two peptides bind to the primary RNA-binding site of the Rho hexamer near its subunit interfaces. In addition, the gene expression profiles of these peptides and Psu overlapped significantly. These peptides also inhibited the growth of *Mycobacteria* and inhibited the activities of Rho proteins from *Mycobacterium tuberculosis*, *Xanthomonas*, *Vibrio cholerae,* and *Salmonella enterica*. Our results showed that these novel anti-Rho peptides mimic the Rho-inhibition function of the ∼42-kDa dimeric bacteriophage P4 capsid protein, Psu. We conclude that these peptides and their C-terminal deletion derivatives could provide a basis on which to design novel antimicrobial peptides.

The Rho-dependent transcription termination plays a major role in the regulation of gene expression in bacteria. The transcription termination of about half of the operons in *Escherichia coli* is controlled by this termination process. The Rho protein, a homohexamer with a protomer of 46.8 kDa, is a highly conserved protein found in most bacteria. It is an RNA/DNA helicase or translocase that dissociates RNA polymerase from the DNA template using its RNA-dependent ATPase activity to bring about the transcription termination (1, 2, 3, 4). It binds to the *rut* site (Rho utilization; a C-rich unstructured region) of the exiting nascent RNA, and this interaction is a prerequisite for its termination function (5). This termination function regulates many physiological processes (4, 6, 7), and the conserved nature of the Rho protein in a wide range of bacteria makes it an ideal target for bactericidal agents.

Psu (polarity suppression) is an unconventional capsid protein of the *E. coli* bacteriophage P4 that moonlights as a specific and efficient inhibitor of Rho (8, 9). It binds and antagonizes Rho *in trans* by creating a mechanical hindrance to the Rho translocation process (10, 11) upon the formation of a V-shaped cap-like knotted homodimer structure at the RNA exit point of the central channel of Rho (11, 12). Its solvent-exposed flexible C-terminal domain (CTD) (helices 6 and 7) (12) interacts directly with Rho, and its N-terminal domain (NTD) imparts stability to the protein (9, 10, 12). Psu is also capable of antagonizing the Rho proteins from different bacterial pathogens (13).

We hypothesize that the Rho-interacting C-terminal region or its derivatives in isolation might show Rho-inhibitory activities, which could be further developed into antimicrobials targeting the Rho protein. Alternative strategies to design new-generation antimicrobials, such as antimicrobial peptides (AMPs), are essential in the wake of the emergence of many multidrug-resistant and extensively drug-resistant pathogenic strains. Efforts to design AMPs from different phage proteins such as endolysins, LysAB2 (14), and PflyF307 (15) have been reported earlier.

Here, we report the design of peptides from the mutagenized CTD (helix 7) of Psu, using a phenotypic screening method. We screened peptides based on their ability to induce growth defects and inhibiting Rho-dependent termination *in vivo*. These peptides not only had the mutagenized sequence from Psu helix 7 but also contained an extra region from the adjacent nucleotide sequence of the plasmid that got appended to their C-terminal region because of frame-shift mutations. *In vitro*, the peptides inhibited the RNA release and ATPase activities of the *E. coli* Rho *via* a direct interaction. The molecular docking and genetic and biochemical evidence revealed that they bind near the primary RNA-binding region of Rho at the interface of its two subunits. Both the peptides and Psu exerted similar genome-wide upregulation upon *in vivo* expressions. The expressions of these peptides caused lethality in *Mycobacteria* and inhibited the *in vitro* functions of the Rho proteins from various other pathogens.

## Results

### A phenotypic screening strategy to design anti-Rho peptides

**Figure 1:**
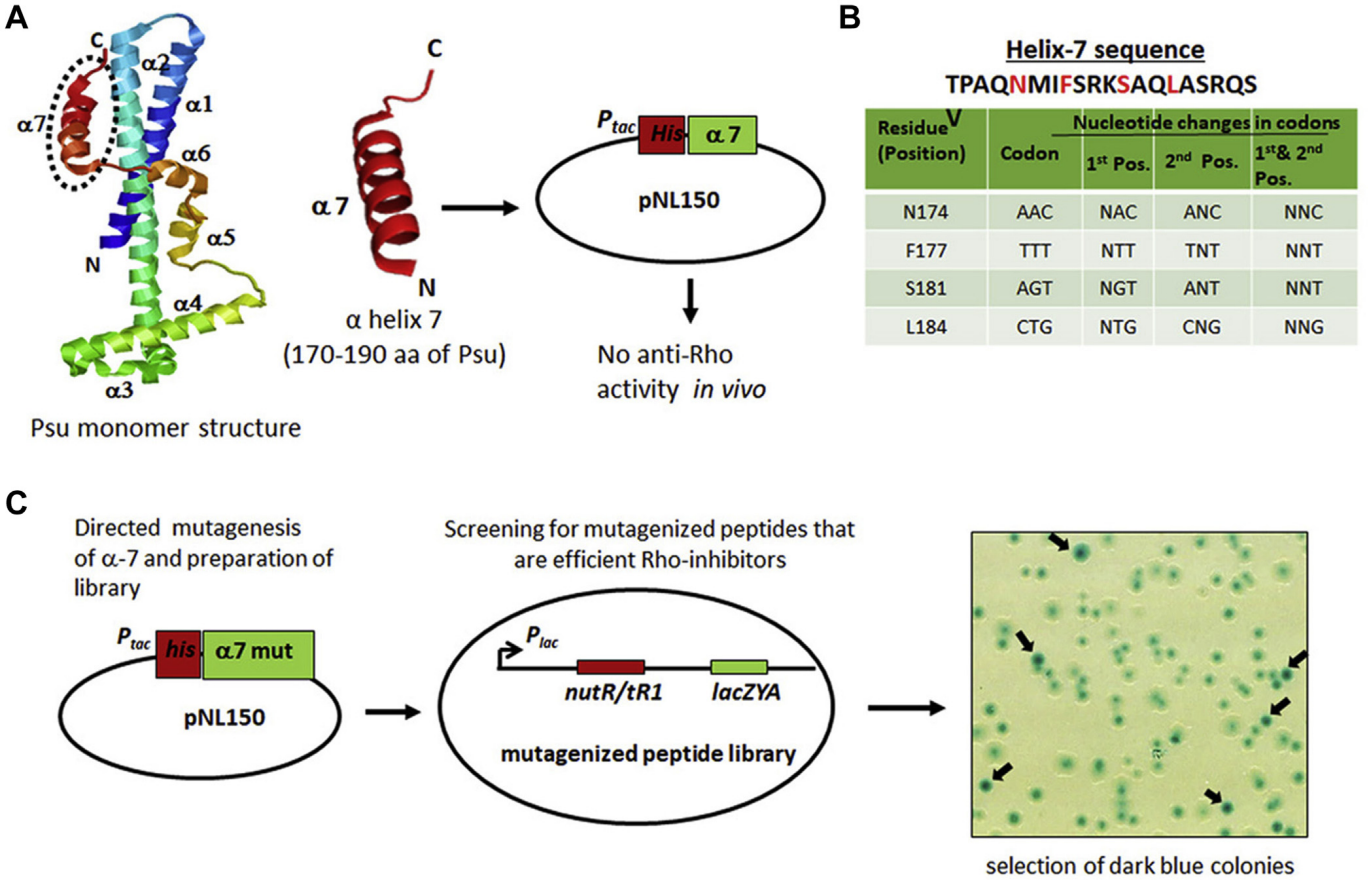
**Design of a phenotypic screening method to screen gain-of-function anti-Rho peptides from the Psu–CTD helix 7.***A*, the Psu monomer derived from Psu dimeric crystal structure (PDB ID: 4DVD) showing the seven helical regions of the protein. The CTD α7 helix is *encircled*. The helix-7 nucleotide sequence was cloned in the pNL150 vector, and upon transformation and expression in MG1655, it did not exert any growth defect. *B*, randomization of codons at the indicated amino acids of helix 7 while designing the oligonucleotides to construct the randomly mutagenized library. N indicates the random nucleotide at the indicated positions. *C*, the mutagenized library was transformed into the MC4100 strain consisting of the lacZYA reporter cassette downstream of a Rho-dependent terminator, *λT*_*R1*_. The gain-of-function peptides containing clones were selected based on the phenotype of *dark blue* colonies on the X-gal LB plates. A scanned image of one such plate is shown. CTD, C-terminal domain.

The bacteriophage P4 capsid protein, Psu, has been shown to act as an inhibitor of the transcription terminator Rho of *E. coli* and various bacterial pathogens (9, 13). The C-terminal helix 7 of the Psu protein (Fig. 1*A*) interacts directly with the Rho (9, 11). Hence, we hypothesized that this helix could be used for developing anti-Rho peptides.

**Figure 2:**
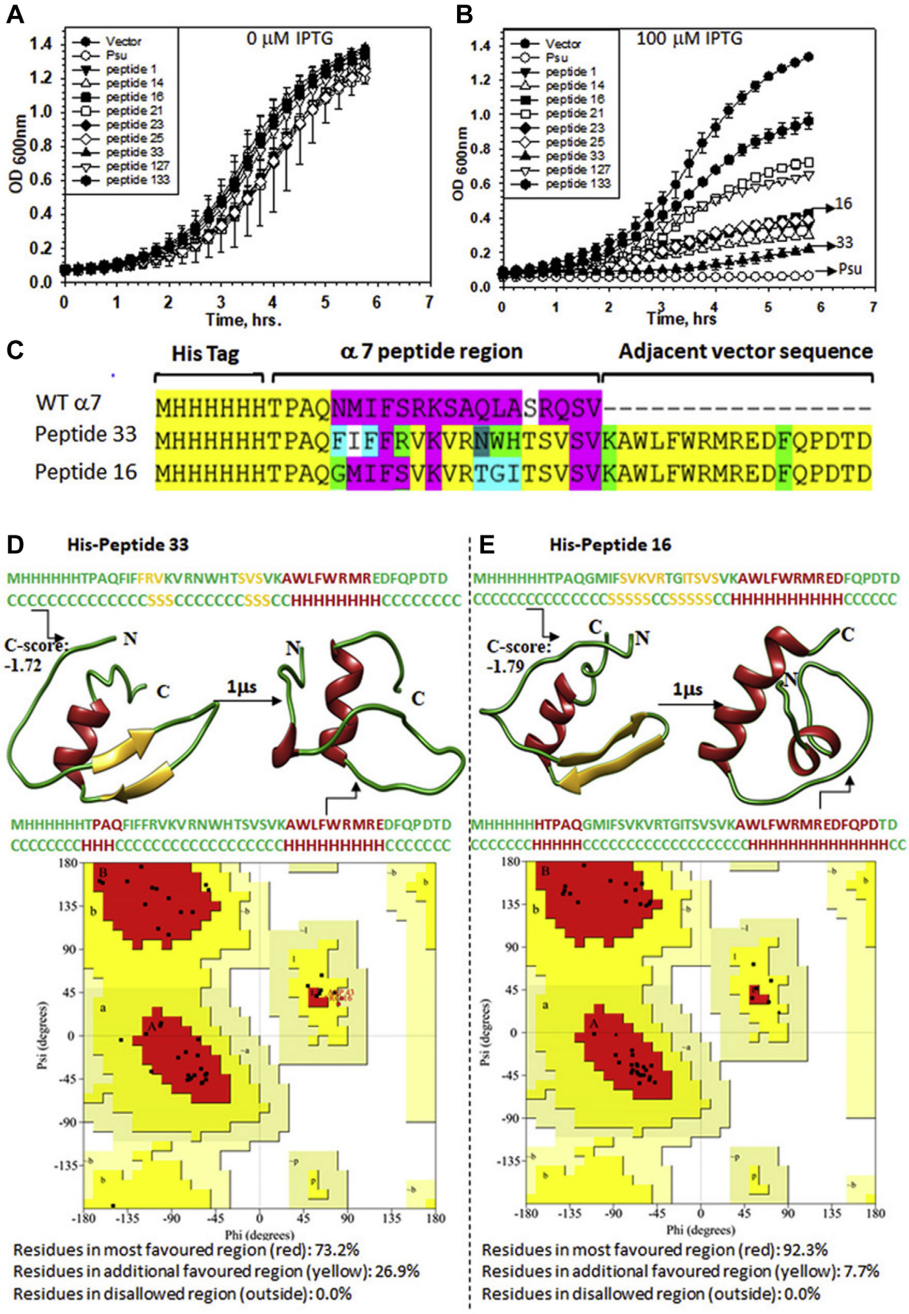
**The anti-Rho peptides.***Panels A* and *B* show the growth curves of the *E. coli* MG1655 transformed with pNL150 vector expressing WT Psu, and different peptides cloned under an inducible *P*_*tac*_ promoter. Growth curves were obtained in the presence of different IPTG concentrations (0 μM [*A*] and 100 μM [*B*]). Error bars represent the SD and were obtained from the measurements of the growth of three independent colonies of each strain. *C*, peptide sequences of two peptides, peptides 16 and 33, that are most effective against the Rho function. A frameshift in the WT Psu-derived peptide sequence led to the addition of adjacent vector sequences at the C-terminal region of the mutated α-7 helix region. *D* and *E*, 3D models of the peptides at the initial time point and after 1-μs molecular dynamics simulation. The initial structure was obtained using the threading methods in the I-TASSER server. The C-terminal sequence of peptides retained their α-helix conformation while the N-terminal region turns into a coil after the simulation. The C-scores of both models are shown. The structure validation of the peptides, the Ramachandran plots, as shown were obtained from the SAVES server. As per the Ramachandran plot validation, both the structures do not have any residue in the disallowed regions after simulation. I-TASSER, Iterative Threading ASSEmbly Refinement; SAVES, Structure Analysis and Verification.

The overexpression of the isolated 21-mer Psu helix 7 did not induce any toxic effect in *E. coli*, unlike the full-length Psu (Fig. 2*A*). Therefore, to obtain gain-of-function peptide(s) from this WT helix 7, we used a phenotypic screening strategy by randomly mutagenizing specific amino acids of this 21-mer peptide. We used different synthetic DNA oligonucleotides containing one or more degenerated codons at the positions corresponding to each of the targeted residues. The residues that were already shown (11) to be important for the interaction with Rho were selected. The amino acid positions of Psu, N174, F177, S181, and L184 were targeted, and the oligonucleotides were designed to mutagenize the first, the second, or both the positions of each of the codon of the targeted residues (Fig. 1*B*). The library of mutant peptides was cloned under the control of an IPTG-inducible *P*_*tac*_ promoter in the pNL150 vector and was expressed in the RS734 strain having a *lacZ*-reporter cassette fused downstream of a Rho-dependent terminator, *tR*_*1*_ (*P*_*lac*_*-nutR/tR1-lacZYA*). In this setup, gain-of-function peptide clones would yield blue colonies on the LB X-gal plates. We screened ∼80,000 colonies. The colonies appearing deep blue were screened and were further checked for their growth-inhibition properties (Fig. 1*C*).

### The anti-Rho peptides

**Figure 3:**
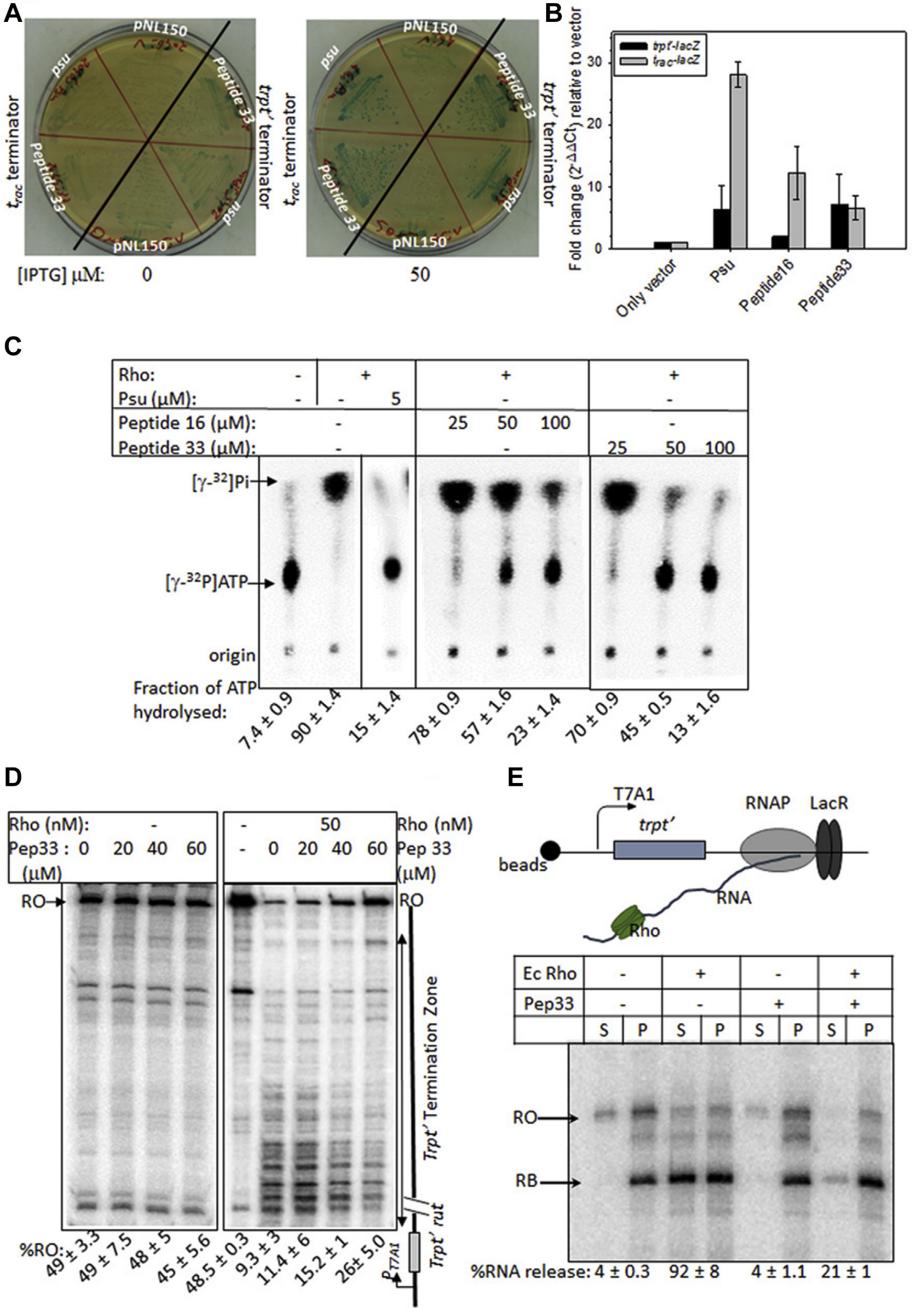
**Inhibition of Rho functions *in vivo* and *in vitro* by the peptides.***A*, *in vivo* Rho-dependent transcription termination assays. MG1655 strains, RS2045 and RS2046, carrying the LacZAY reporter cassette fused downstream of the *trpt*′ and *t*_*rac*_ terminators, respectively. These strains upon transformations with the pNL150 vector expressing Psu or the peptides were streaked on LB–X-gal plates supplemented with indicated amounts of IPTG. Appearances of the *blue* colonies indicated that the Rho function is inhibited. *B*, qRT-PCR assays of the expressions of the LacZ gene fused downstream of the indicated terminators. The fold changes in C_t_ values relative to that obtained in the presence of the empty vector gave a quantitative measure of the LacZ expressions as well as the *in vivo* inhibition of the Rho-dependent termination by Psu and peptides 16 and 33. SDs were obtained from at least three biological replicates. *C*, *in vitro* inhibition of ATPase activities of Rho. The fractions of ATP hydrolyzed by Rho in the presence or absence of indicated concentrations of the peptides and Psu are shown in the autoradiograms. *λtR1* RNA was used as the substrate for the Rho. The SDs were calculated from two to three measurements. The spliced parts of the figure are indicated by *vertical lines*. *D*, single round *in vitro* Rho-dependent transcription termination assays using a linear DNA template, where transcription is initiated from the T7A1 promoter and elongated through the *trpt*′ termination zone. Terminated transcripts are seen in the absence of peptide 33. The termination zone was shifted downstream, and the run-off (RO) products were visible in the presence of the peptide. Concentrations of Rho and the peptide are indicated. Fractions of RO was calculated as [RO]/[all the terminated products + RO]. SDs were calculated from 2 to 3 measurements. *E*, cartoon of an EC stalled at the template with T7A1-trpt′ terminator: This DNA template is immobilized onto magnetic beads *via* a biotin linkage at the 5′ end. The transcription is initiated from a T7A1 promoter and the EC is roadblocked (RB) at a lac repressor (LacR) bound at the lac operator sequence downstream of the *trpt*′ terminator. Rho, peptide or Rho–peptide complexes together with ATP were added to the stalled EC. The autoradiogram showing RNA release by Rho in the S fractions (half of the supernatant) and the P fractions (the other half of the supernatant and the whole of the pellet; S + (S + P)) in the presence and absence of peptide 33. RO indicates a run-off product. The fraction of RNA released was calculated as {2S/[S + P]}, and the SDs were obtained from 2 to 3 measurements. C_t_, threshold cycle; EC, elongation complex; qRT-PCR, quantitative reverse transcription PCR.

The mutant clones obtained above were expressed in a WT MG1655 strain in the presence of 100 μM of the inducer, IPTG, and the growth assays were followed (Fig. 2, *A* and *B*). We observed significant growth defects upon expressions of several peptide clones. Among the nine peptide clones, peptides 16 and 33 exhibited severe growth defects and strong *in vivo* antitermination of Rho-dependent termination (Fig. 3). The *in vivo* Rho inhibitory properties of these two peptides were comparable with that of Psu.

Upon nucleotide sequencing of these peptide clones, we observed that all the peptides, in addition to the desired point mutations, acquired novel sequences, which evolved because of a frameshift mutation in the helix-7 sequence (Figs. 2*C* and S1). The addition of new sequences was from the adjacent vector sequences at their C-terminal region, which converted these gain-of-function peptides into 38-mer peptides from the original 21-mer helix 7 of Psu.

*In silico* modeling of these two peptides, using the threading method in the Iterative Threading ASSEmbly Refinement (I-TASSER ) server, revealed that the C-terminal new adjacent sequence was predicted to fold into a helical structure, whereas a part of the N-terminal region formed weak antiparallel β-sheets that were stabilized as random coils after a 1-μs molecular dynamics (MD) simulation (Fig. 2, *D* and *E*, and Fig. S2). This adjacent sequence might be responsible for structurally stabilizing the peptides *in vivo* that were absent in the 21-mer WT helix 7 rendering the latter unstable and nonfunctional. The quality of the predicted structures was high with a confidential score for peptides 16 and 33 of −1.79 and −1.72, respectively. The structure validation by the Ramachandran plot showed that both the peptides have no residues in the disallowed regions (Fig. 2, *D* and *E*).

To understand the structural basis of the variations in their functional efficiencies, we modeled and performed dynamic simulations of all the other peptides and compared their RMSD structural deviations from peptide 33 (Fig. S1*B*). We observed that the changes in the amino acid sequences in the helix-7 regions induced structural variations ranging from ∼6 Å to ∼11 Å as compared with peptide 33. Unlike peptides 16 and 33, in most of the other peptides, the N-terminal β-sheets remained intact after the MD simulation. Also, the spatial orientations of the C-terminal helixes of these other peptides were significantly different compared with peptide 33. We reasoned that these structural changes could have affected the functions of these peptides.

CD spectra of peptides 16 and 33 also revealed that they are predominantly α-helical (Fig. S3).

### Anti-Rho functions of the peptides

We tested the *in vivo* anti-Rho function potentials of peptides 16 and 33. For this purpose, we first performed *in vivo* Rho-dependent termination assays using an MC4100 strain having a single-copy *galEP3* reporter in the chromosome. This reporter cassette consists of a series of Rho-dependent terminators present in the *IS2* element inserted at the beginning of the galactose operon. If the expression of Psu or the peptides (cloned in pNL150, under an IPTG-inducible promoter, *P*_*tac*_) inhibits the Rho function, antitermination will occur through these terminators, leading to expression of the galactose operon that would be manifested as red or pink colonies on MacConkey–galactose indicator plates. We observed the appearance of red colonies upon overexpression of the WT Psu and the peptides indicating the inhibition of Rho-dependent termination *in vivo* (Fig. S4*A*).

Next, we monitored the effects of the *in vivo* expressions of Psu and the peptides on Rho-dependent termination at two well-known terminators, *trpt*′ and *t*_*rac*_, using the *lacZ* reporter system. They are *P*_*lac*_*-trpt*′*-lacZAY* (RS2045) and *P*_*lac*_*-H19B nutR/t*_*R1*_*-lacZAY* (RS2047), where the terminators are fused upstream of the *lacZ* genes and are inserted in the chromosome of *E. coli* MG1655 *Δrac Δlac.* The colonies would appear blue on the LB–X-gal indicator plates if the Rho-dependent termination is inhibited. We observed that upon expressions of Psu and the peptides (induction by 50 μM IPTG), deep blue colonies appeared on the indicator plates. Colonies were white or pale blue in the presence of an empty pNL150 vector (Fig. 3*A*). We repeated the *in vivo* termination assays using quantitative reverse transcription PCR (qRT-PCR) to get more quantitative data by measuring the level of lacZ expression using the same terminator-*lacZAY* fusion templates described above (Fig. 3*B*). The strains described in Figure. 3*A* having these two *lacZ* fusions in the chromosome and harboring the pNL150 vector alone or expressing Psu or the peptides were used. Upon induction by IPTG, the expression levels of the *P*_*lac*_*-t*_*rac*_*-lacZ* fusion were observed to be enhanced by 28-fold when Psu was expressed, whereas in the presence of the peptides, this level was increased by 6- to 12-folds. In the case of the *trpt*′*-lacZ* fusion, these enhanced levels of expression were less but still were significant. These results further confirmed that like Psu, peptides are capable of inhibiting Rho-dependent termination *in vivo* efficiently.

Earlier, we have shown that *in vitro,* the binding of Psu to Rho reduced the rate of RNA-dependent ATP hydrolysis of the latter (9, 13). We used an *in vitro*–synthesized RNA having the *λtR1* terminator sequence to induce the *in vitro* ATPase function of the *E. coli* Rho. We observed that like Psu, both the peptides inhibited the ATPase activity of the *E. coli* Rho (Fig. 3*C*). Owing to higher affinity, peptide 33 was more efficient than peptide 16 in inhibiting this Rho function *in vitro*. However, about 10 times more molar amount of peptides than Psu was required to observe this inhibitory activity. The requirement of a higher concentration of peptides to exert their effect is consistent with the requirement of a higher level of IPTG induction in the *in vivo* experiments. It should be noted that the peptides used in the *in vitro* studies were chemically synthesized.

Psu is capable of inhibiting *E. coli* Rho-dependent termination in an *in vitro*–purified system (9). Next, we assayed the peptide-mediated inhibition of the *in vitro* transcription termination functions of the Rho. We used a linear DNA template where a *trpt*′ terminator is fused downstream of a strong T7A1 promoter. On this template, efficient transcription termination over a terminator zone is usually observed in the presence of Rho (Fig. 3*D*; (16)). The amount of run-off transcripts at the end of this template gives the measure of Rho inhibition by the peptides. We observed that the amount of run-off transcripts increased to ∼28% at higher concentrations of peptide 33 from ∼6% in its absence. A similar level of *in vitro* Rho inhibition was earlier observed in the presence of Psu (9). Peptide 33 did not have any effect on transcription when Rho was absent (lane 2 from left). The effect of peptide 16 in this type of transcription termination assays was observed to be much less, which could be due to its weaker affinity for Rho (data not shown).

Next, we measured the inhibition of Rho-mediated RNA release from a stalled elongation complex (EC) by the peptides. We designed a setup where the EC is stalled on a template bound to magnetic beads at a particular position inside the *trpt*′ terminator region using the lac repressor as a roadblock (RB) (Fig. 3*E*, top panel). On this template also, the transcription is initiated from the T7A1 promoter. In this setup, the RNA released because of the Rho-dependent transcription termination would be visible in the supernatant (S). We observed that in the presence of either Psu (Fig. S4*B*) or the peptides (Fig. 3*E* for peptide 33 and Fig. S4*B* for peptide 16), the Rho-induced RNA release from the stalled EC was significantly inhibited. Peptide 16 could efficiently inhibit the RNA release of Rho only when stalled EC was used (Fig. S4*B*). Similar to the ATPase assays, higher concentrations of peptides than Psu were required to bring about this inhibition function. Peptide 33 exerted its action at a lower concentration than peptide 16, indicating a tighter binding to Rho by the former (Fig. S4*B*). It should be noted that the peptides do not have any adverse effect on the *in vitro* transcription process in the absence of Rho (Fig. 3*E*, no Rho lane). In general, at ≥50 μM, peptide 33 exerted its maximal effects. However, as these peptides were synthesized and commercially purchased, the concentration requirements to exert the maximum effect varied between different lots.

These observations strongly indicate that the peptides are capable of inhibiting the *in vitro* functions of Rho protein in a manner comparable with that observed for the WT Psu.

### Peptide–Rho interactions

**Figure 4:**
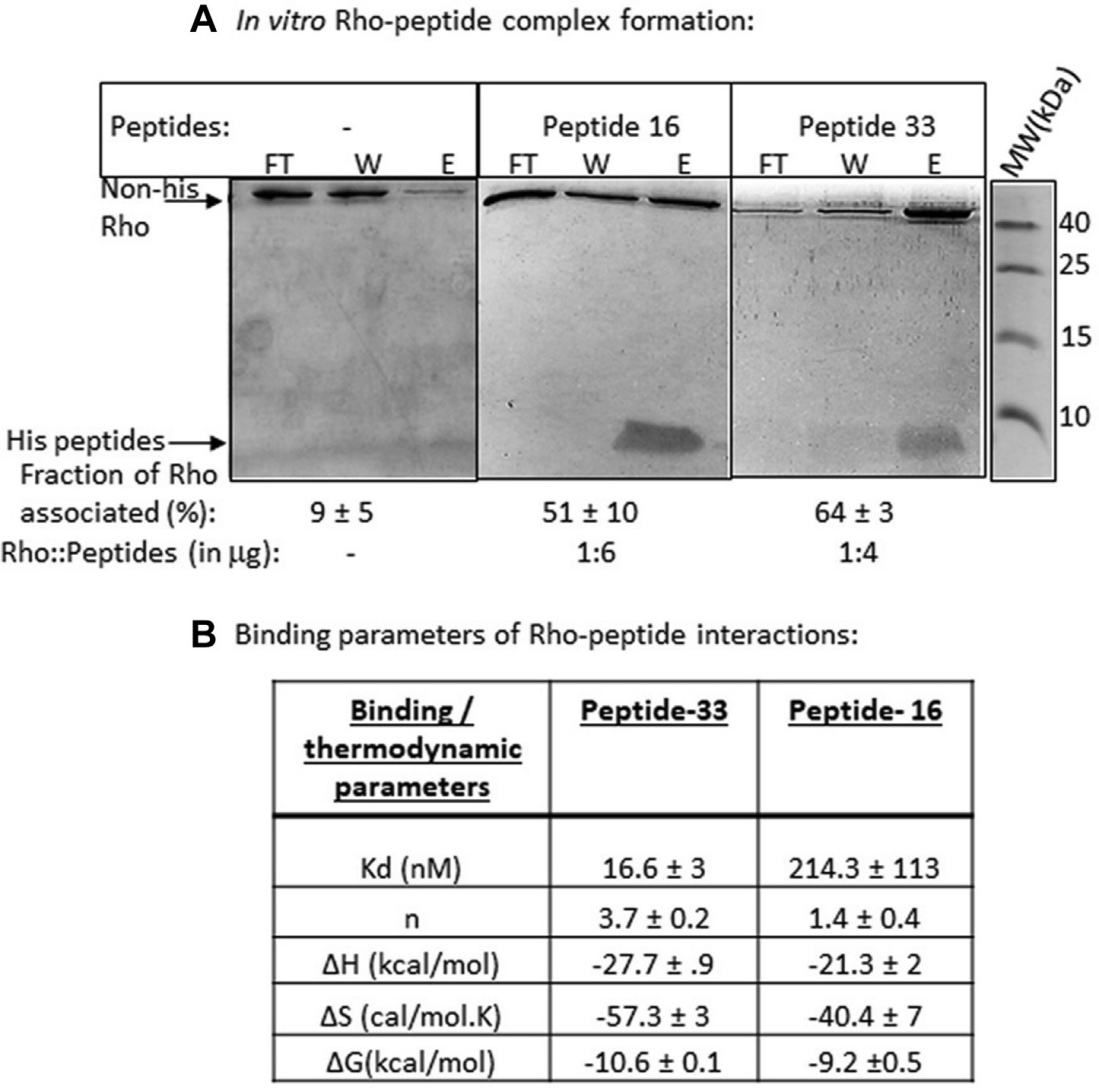
**Interactions of peptides and Rho.***A*, complex formation by peptide 16 and peptide 33 with P167L Rho by *in vitro*, pull-down assays. Mixtures of Rho and His-tagged peptides were added to Ni-NTA beads, and the flow-through (FT), wash (W), and the elute (E) fractions were collected. The complexes were seen to be formed in the eluted fractions. Rho and the peptide bands are indicated. Fractions of Rho-associated peptides were calculated as follows: [E]/([FT] + [W] + [E]). The ratios were calculated from the amounts of Rho or peptides expressed in micrograms. SDs were calculated from two or three independent measurements. The molecular weight markers are aligned next to the gel. *B*, the table listing the dissociation constants (*K*_d_), binding stoichiometry (n), changes in the enthalpy (ΔH), entropy (ΔS), and free energy (ΔG) for the interactions between Rho and the peptides. These parameters were obtained using ITC methods. All values represent an average of three independent experiments and the SDs. ITC, isothermal titration calorimetry.

Earlier, we have demonstrated the formation of a specific Psu–*E. coli* Rho complex *in vivo* by overexpressing both the proteins together in the same strain (9). However, we failed to obtain a stable *in vitro* Rho–Psu complex with the WT Rho. We observed that an *in vitro* stable complex between Psu and Rho could form if a mutant Rho, P167L, was used (11). This mutant Rho was obtained as a suppressor of a Psu mutant that was defective in binding to WT Rho (11). This Rho mutant appeared to form a stable hexamer at a lower concentration unlike the WT Rho, which could have improved its affinity for Psu. We assumed that this Rho mutant would also have a higher affinity for the peptides. Hence, to demonstrate peptide–Rho interactions *in vitro*, we have used P167L Rho. We performed the pull-down assays by using His-tagged versions of Psu (Fig. S4*C*) and peptides 16 and 33 (Fig. 4*A*) and the non–His-tagged P167L Rho, where peptides and Psu were bound to nickel nitrilotriacetic acid (Ni-NTA) agarose beads. In this assay, unbound Rho would be visible in the flow-through and the wash fractions, and the bound Rho would be in the eluted fraction. In all these cases, ∼ 40 to 70% of Rho proteins were found to be associated with the peptides (Fig. 4*A*), a level comparable with what we observed for the Psu–Rho complex (Fig. S4*C*). The complexes were observed to be formed at the ratios of Rho hexamer:peptide 16::1:6, Rho hexamer:peptide 33::1:4, and Rho hexamer:Psu:: 1:3. In all these cases, ratios were calculated from the amounts of Rho/Psu/peptides expressed in micrograms (Fig. 4*A*). These results indicated a direct interaction of the peptides with Rho, which induced the anti-Rho functions.

To understand the mode of interactions of the peptides with P167L Rho, we performed the isothermal titration calorimetry (ITC) analysis of peptides 16– and 33–Rho interactions. The binding and thermodynamic parameters are listed in Figure. 4*B*. We observed the following. (1) Peptide 33 binds to P167L Rho with a fairly high affinity (*K*_d_ = 17 nM) with a Rho hexamer:peptide 33::1:4 binding stoichiometry. Peptide 16 binds with much weaker affinity and with significantly reduced stoichiometry. This weaker binding of peptide 16 is consistent with its requirement of higher concentrations in the functional assays (Fig. 3). (2) The thermodynamic parameters indicated that the binding of both the peptides is highly enthalpy driven, most likely because of the formation of specific H-bonds between the amino acids of the peptides and Rho (17), which is common for smaller peptides fitting into specific pockets of larger macromolecules *via* an “induced-fit” mechanism.

### The functional importance of regions of the peptides

The aforementioned results showed that the functional peptides contain an N-terminal His-tag sequence and an additional sequence from the vector appended to their C-terminal region in addition to that derived from Psu helix 7. To understand the importance of additional N- and C-terminal regions, we made a series of deletions in these regions and performed growth assays in similar ways as described in Figure. 2*A*.

**Figure 5:**
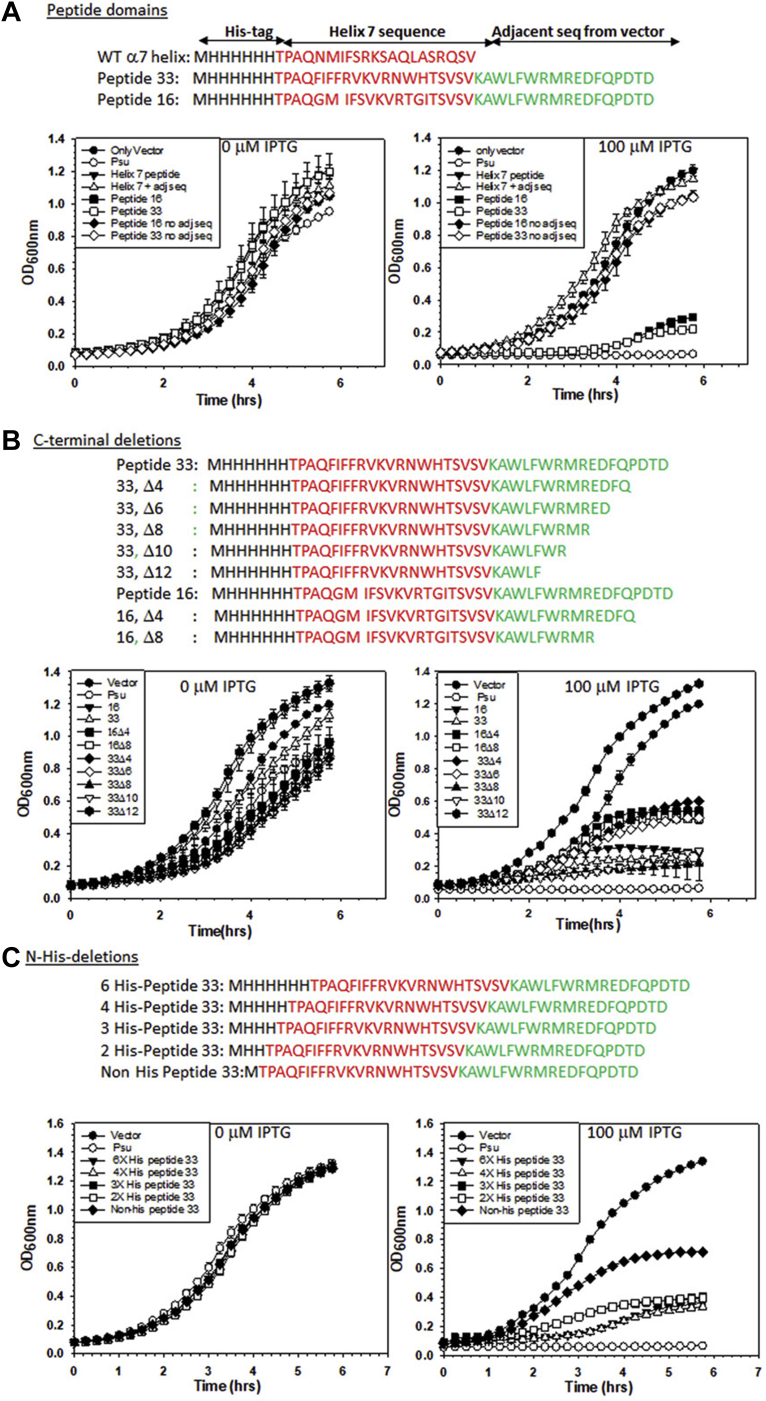
**Effects of different deletion derivatives of the peptides on the growth curves.***A*, peptide domain deletions. Growth curves of the MG1655 strains expressing full-length and different adjacent vector sequence derivatives of the indicated peptides. WT helix-7 sequence alone and together with the adjacent vector sequence were also included in the analyses. WT Psu and the empty vector plasmids were used as controls. The secondary cultures of the overnight culture of all the strains were induced with different IPTG (0 μM and 100 μM) concentrations to check the growth kinetics. *B*, growth curves of the same strains expressing indicated C-terminal derivatives of peptides 33 and 16 obtained in the presence and absence of 100 μM IPTG. *C*, growth curves of the same strains expressing indicated N-terminal His-tag sequence deletions of peptide 33 obtained in the presence and absence of 100 μM IPTG. In all the cases, SDs were obtained from three independent measurements from three colonies of each strain.

We observed that the N-terminal 6XHis-tagged WT Psu helix 7 sequence either in the presence or absence of the C-terminal adjacent vector sequence did not cause a growth defect in the presence of IPTG (Fig. 5*A*; see the plots, helix 7 peptide, and helix 7 + adjacent sequence). The presence of C-terminal vector sequences (shown in green) in peptides 16 and 33 was found to be essential for their function *in vivo* (Fig. 5*A*). This suggests that in addition to the C-terminal vector sequence, the mutations present in the helix-7 region (shown in red) of peptides 16 and 33 are also important. The vector sequence might be providing structural stability to the mutated helix-7 region of the peptides.

The length of these two peptides is too long to be functional as AMPs as cells would not allow their entry when added exogenously. So, we explored the minimal length of these peptides required to remain functional. We constructed a series of truncated versions of the peptides with the deletions of amino acids from their C-terminal region and expressed them *in vivo* in the presence of IPTG and monitored their abilities to induce growth defects in a similar way as described in Figure. 2 (Fig. 5*B*). We observed that up to eight amino acids could be deleted from the C-terminal region of peptide 33, without incurring significant functional defects, whereas deletions of 10 and 12 amino acids caused a slight loss of its function. Interestingly, the deletion of eight amino acids of peptide 33 appeared to induce more growth defects than its full-length counterpart. However, four and eight amino acid deletions from the C-terminal region of peptide 16 caused partial loss of function. *In vivo* termination assays at the *trpt′* and *t*_*rac*_ terminators using qRT-PCR assays revealed that the deletion of eight or ten amino acids induced more than 5-fold and 3-fold enhancement of expressions of the *lacZ* fused to these two terminators, respectively (Fig. S4*D*). This indicates that these deletions did not affect their anti-Rho–dependent termination properties *in vivo.* The above results also suggested that in addition to imparting structural stability to the peptides, the first nine amino acid sequences (KAWLFWRMR, Fig. 2*B*) of the adjacent vector region might take part in direct interactions with Rho. This smaller peptide might serve as a better template for designing AMPs.

All the screened peptides have 6X-His-tag sequences, which might influence their functions. To explore their role(s), we constructed sequentially deleted versions of the N-terminal 6XHis-tag sequences of both the full-length peptide 33 (Fig. 5*C*) and peptide 33Δ8 (Fig. S5*A*). These peptide 33 derivatives were expressed *in vivo*, and the same growth assays described above were performed. We observed that the inhibitory effects were not significantly reduced when 2, 3, and 4 histidines were deleted, whereas the non-His derivative of peptide 33 exhibited partial growth inhibition. The absence of up to four histidine from the N-terminal 6X-His sequence did not affect the function of peptide 33Δ8 either. This indicated that the presence of one or two histidine amino acids at the N-terminal region of peptide 33 might improve its stability and/or solubility *in vivo*. This notion was reinforced by the fact that the non-His peptide 33 when synthesized chemically showed very poor solubility in aqueous solvents (data not shown). Only the full-length N-terminal His-tag peptides 16 and 33 had good solubility in water, and hence, we did not attempt to perform *in vitro* assays with other deletion derivatives.

### Mode of interaction with Rho

Earlier, we have proposed that the Psu dimer forms a cap-like conformation over the Rho central channel, thereby blocking the latter's translocation process (11). The two interacting regions of Psu on the Rho protein were identified around 144 to 153 and 347 to 353 amino acids, and the Rho point mutants, R144E, R146E, and, E148R from one of these binding regions did not bind to Psu (11). To identify the peptide–Rho interaction surface on the latter, we first tested whether the above Psu-specific Rho-mutants are also refractory to binding the peptides. In similar growth assays as described in the earlier sections, we observed that the presence of the Rho mutants, R144E, R146E, and E148R, could not prevent the peptide-induced growth defect unlike Psu (Fig. S5, *B–D*). So, peptides 16 and 33 may not bind to the Psu-binding regions of Rho, which is not unusual because of the smaller structures of the peptides that enable them to fit in many other region(s) of a big macromolecule such as Rho offering many binding pockets.

**Figure 6:**
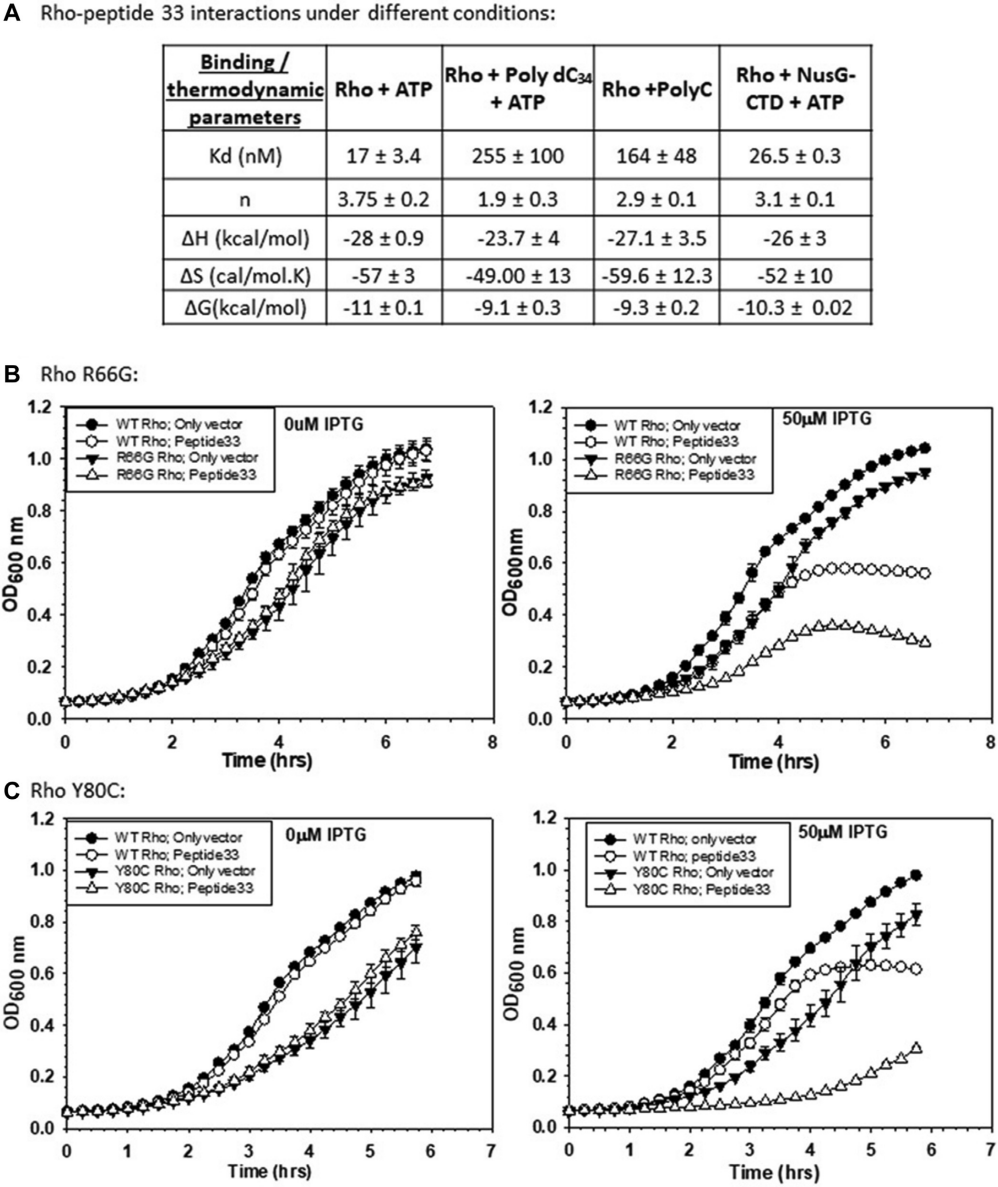
**Mode of Rho–peptide 33 interactions.***A*, the table lists the binding and the thermodynamic parameters of Rho–peptide 33 interactions in the presence or absence of indicated Rho-binding factors. All the values are an average of three independent experiments with SDs. *Panels B* and *C* depict the growth curves of MG1655 strains having either the WT or the Rho mutants, Y80C and R66G, expressing the indicated peptides in the presence of 50 μM IPTG. SDs were obtained from three independent measurements.

To probe the mode of interaction of the peptides with Rho, we carried out Rho–peptide binding studies using ITC in the presence of different bona fide Rho-binding factors. We reasoned that in case the presence of any one or more of these factors reduced the affinity of Rho for the peptides, it is likely that the peptide-binding site(s) overlaps with that of the factor. We performed these assays in the presence of (i) polydC_34_ that binds to the N-terminal primary RNA-binding site (PBS), (ii) polyC that binds to both the PBS and the secondary RNA-binding site (SBS) simultaneously, and (iii) NusG CTD that binds in a hydrophobic patch in the region encompassing 203 to 221 amino acids (16, 18). We observed that the affinity of peptide 33 for Rho was reduced by 15-fold and 10-fold in the presence of polydC_34_ and polyC, respectively, whereas it was not affected significantly when NusG CTD was present (Fig. 6*A*). The binding stoichiometry of peptide 33 was also reduced when Rho was bound to the single-stranded DNA and RNA. However, thermodynamic parameters were not changed significantly, which indicates that the DNA and RNA species offer competitive inhibition to peptide 33. These results strongly suggest that the interaction site(s) of peptide 33 might be in the vicinity of the N-terminal PBS of Rho. Interestingly, peptide 33 was unable to interact with an isolated Rho–NTD monomer (data not shown), which indicates that Rho hexameric assembly is essential and it is likely that the subunit interfaces at the N-terminal region could play important role in the binding.

**Figure 7:**
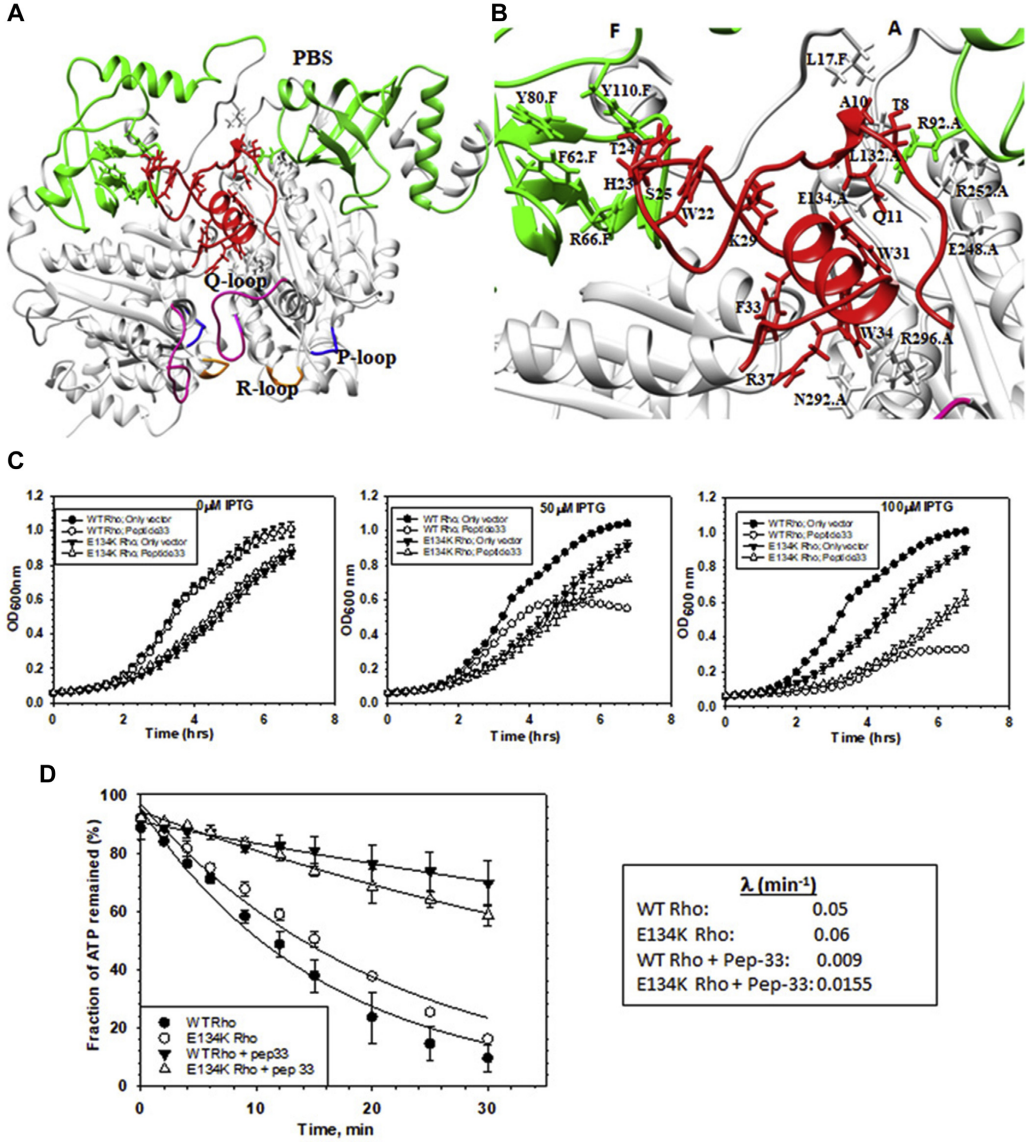
**Interacting region(s) of Rho for peptide 33.***A*, molecular docking of peptide 33 onto the dimer of Rho. Peptide 33 (*red*) bound at the interface of the two chains, A and F, of Rho in its primary RNA-binding site (PBS, *green*). The PBS (22–116, *green*), P-loop (179–183, *blue*), Q-loop (278–290, *magenta*), and R-loop (322–326, *orange*) from each chain of Rho are highlighted. *B*, an amplified view of the peptide–Rho interface. The key residues of Rho and the peptide obtained from the Hot spot prediction are displayed. *C*, the growth curves of MG1655 strains with WT or E134K mutant Rho upon expression of peptide 33 in the presence of indicated concentrations of IPTG. Strains with empty vectors were used as controls. SDs were obtained from three independent measurements from three colonies of each strain. *D*, time courses of ATP hydrolysis of WT and the E134K Rho mutant in the presence and absence of peptide 33. ATP hydrolysis was induced in the presence of an *in vitro*–synthesized RNA having the *λT*_*R1*_ terminator sequences. Average rates are described in a tabular form. SDs shown in the curves were calculated from two to three measurements. The average rates were calculated from the curve-fitting of the points shown in the plots to an exponential decay equation of the form: y = A exp(−λt), where λ is the rate and A is the amplitude.

The aforementioned binding data indicated that peptide 33 binding affects the primary RNA-binding properties of Rho. Hence, we reasoned that the presence of peptide 33 would cause synthetic defects with the Rho mutants, R66G and Y80C, which are defective for primary RNA binding (16) (see Fig. 7*B*, for their locations in the PBS). This defect is expected to be manifested as enhanced inhibition of growth. We followed the growth curves of the WT and these Rho mutants in the presence and absence of the expression of peptide 33 (Fig. 6, *B* and *C*) in a similar way as described in Figure. 2. We observed that compared with WT, the effects of peptide 33 on the growth curves of the Rho mutants were very severe (even in the presence of 50 μM of IPTG), which indicates that peptide 33 exerted synthetic growth defects in the presence of the Rho–PBS mutants. This functional data further support the proposition that peptide 33 binding to Rho affects RNA binding at the PBS of Rho.

### Interaction site(s) of the peptides on Rho

The *in silico* methods of small-peptide docking onto proteins are quite reliable. We used the predicted structure of the His-peptide 33 (Fig. 2) and the solved 3D structure of the Rho hexamer (PDB ID: 3ICE) for the docking regimen. The Rho–His peptide 33 docking was performed in the ClusPro server. This server produced 25 clusters with a single complex in each. Among them, we selected a representative structure (center and lowest energy) from cluster 0 that has the highest cluster size of 98. In this peptide 33–Rho complex, the peptide was observed to be bound in between the A and F chains (Fig. 7, *A* and *B* and Fig. S6*A*) in the vicinity of the PBS in the NTD and also close to the Q-loop of the SBS of the Rho hexamer.

We performed MD simulation up to 200 ns to study the stability of peptide 33 in the binding region of Rho and to understand the mode of peptide interactions with Rho (Fig. S7). As evident from Figure S7*A*, the RMSD reached equilibrium at ∼30 ns and remained stable throughout the simulation period. The compactness of the complex was also maintained throughout the simulation as is reflected in the RMS fluctuation plot (Fig. S7*B*). The free-energy landscape analyses to retrieve the representative structure in Figure S7, *C* and *D* indicated the existence of the single lowest energy cluster. This simulation suggests that the peptide remained stable in the binding pocket at the interface of the A and F chains of Rho during the simulation period.

The hot spot residues present in the Rho–peptide 33 interface that have the highest specificity and affinity for their binding partners were identified using the Knowledge-based FADE and Contacts (KFC) and HotRegion servers. The KFC server implements machine learning modules (KFC2A and KFC2B), whereas the HotRegion database identifies “hot region” by combining the residue network topology with residue energy profile-based clustering approaches. A residue was considered as a “hot spot” only when it was predicted by both these servers. The key hot spot residues involved in the interactions at the interface of Rho protomers (chains A and F) and in peptide 33 are tabulated in Figure S6*D*, and some of them are highlighted in Figures 7*B* and S6*C*.

This modeling of the Rho–peptide complex revealed that Rho-PBS amino acids Y80 and R66 come close to peptide residues 24 and 25 (Fig. S6*B*), which could be the reason for synthetic defects caused by the mutations in these two positions in the presence of peptide 33 (Fig. 6, *B* and *C*). It should be noted that the location of P167L mutation, which has a higher affinity for Psu/peptides, is not close to the peptide-docking site(s) (Fig. S6, *B* and *D*). It is located at the interface of the subunits near the R-loop, enabling a more stabilized oligomeric structure of Rho, which might have increased the affinity for Psu/peptides.

To further validate the functional significance of this modeled complex, we chose the Rho mutant, E134K, as this residue comes within interacting distances of several residues of the docked peptide 33 (Fig. S6*C*), and tested whether it causes a defect in the Rho–peptide interaction both *in vivo* and *in vitro.* We performed the growth assays of the WT and E134K Rho mutant strains in the absence and presence of peptide 33 (Fig. 7*C*). We observed that the induction of the peptide caused a less severe effect on the growth of the E134K Rho than its WT counterpart. We then followed the *in vitro* rate of ATPase activities of the WT and the E134K Rho proteins in the presence and absence of peptide 33 in the same way as described in Figure. 3*C* (Fig. 7*D*). The rate analyses revealed that the presence of peptide 33 reduced the rate of ATP hydrolysis of the WT Rho by ∼5.5 fold, whereas the rate was reduced to ∼3.75 fold in the case of the E134K Rho. These results indicated that under *in vitro* conditions, the binding of peptide 33 is partially affected by a mutation at the E134 position of Rho. This finding further reinforced the proposition that peptide 33 could bind in the PBS at the interface of the two monomeric chains (A and F) of the Rho hexamer.

### Genome-wide effects of the anti-Rho peptides

**Figure 8:**
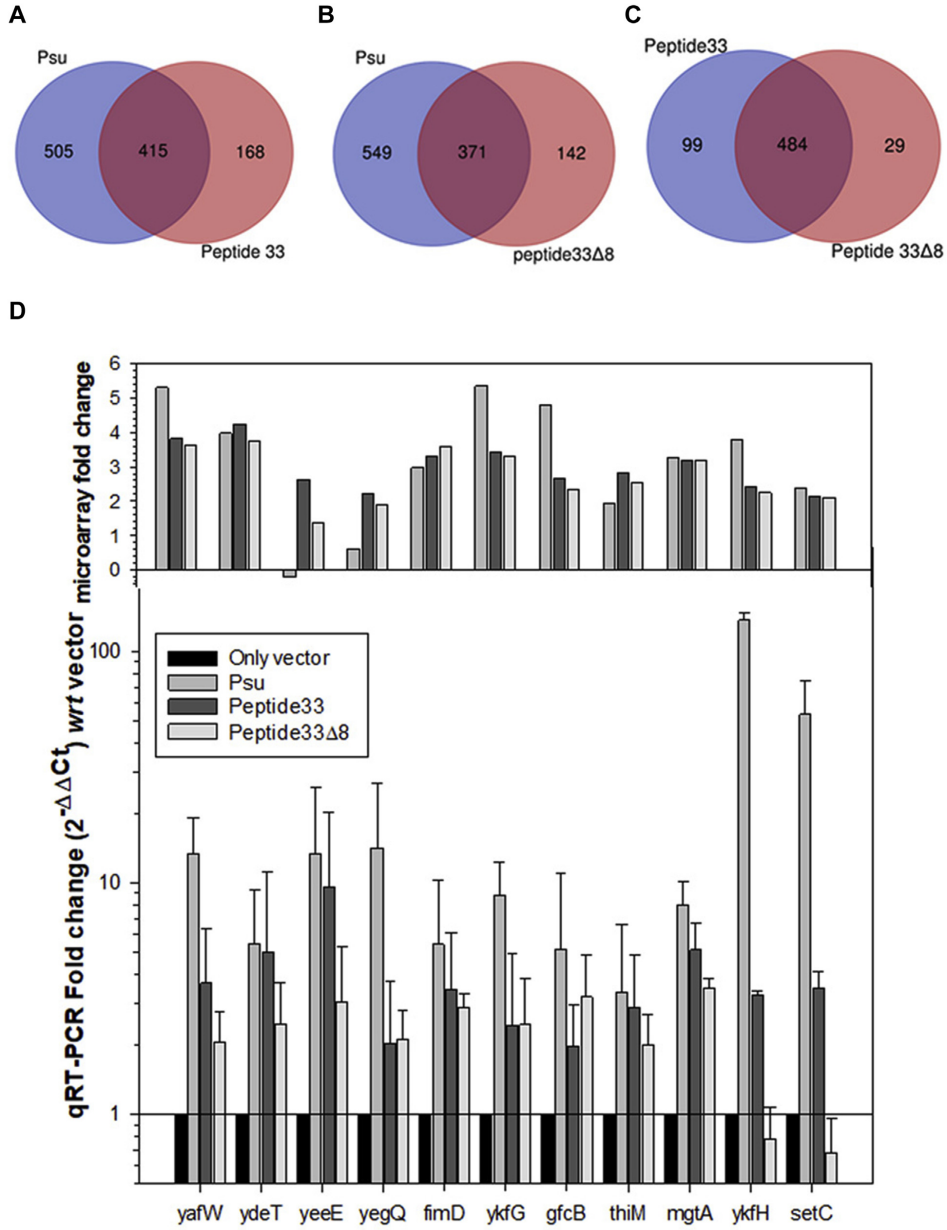
**Genome-wide effects of the peptides and Psu.** Venn diagram representing the upregulated genes obtained from microarray analyses common in between the strains expressing (*A*) peptide 33 and Psu. *B*, peptide 33CTDΔ8 and Psu and (*C*) peptide 33 and peptide 33CTDΔ8. *D*, qRT-PCR analyses of few upregulated genes in the presence of Psu, peptide 33, and peptide 33CTDΔ8 to validate the microarray data. Above the fold-change values of C_t_, microarray fold-change data for the corresponding genes are shown. Fold changes were obtained relative to the values obtained in the presence of the empty pNL150 vector. All the C_t_ values are described in Table S1. SDs were obtained from the measurements of at least three biological replicates. C_t_, threshold cycle; qRT-PCR, quantitative reverse transcription PCR.

The inhibition of Rho function *in vivo* elicits a genome-wide upregulation of various genes (19). To observe genome-wide effects due to the expressions of Psu or the peptides, we performed microarray analyses of MG1655 strain expressing WT Psu, peptide 33 and peptide 33CTDΔ8, and empty vector of the pNL150 plasmid and compared the fold change in gene expressions of the peptides/Psu expressing strains relative to that having the empty vector (Fig. 8). Upon expressions of Psu and the peptides, we observed that a significant number of affected genes that were upregulated are expressed in the mid-log phase, which is common for both Psu and peptide 33 or peptide 33CTDΔ8 (Fig. 8, *A* and *B*). Hence, the peptides may affect many similar physiological pathways as affected by Psu. However, there are also some unique genes upregulated in all cases. Considerable overlapping between the genes affected by the two different peptides was also observed (Fig. 8*C*). To validate the microarray data, we performed qRT-PCR assays of few genes that showed upregulations in the microarray data (Fig. 8*D*). Most of the genes showed significant upregulation in the presence of Psu or the peptides. The highest level of upregulations of the tested genes was observed in the presence of Psu. The presence of peptide 33 produced a higher level of upregulation of these genes than that when its CTDΔ8 derivative was expressed.

The comparison of the genes affected by both the peptides and those affected in the presence of a defective Rho mutant N340S (an SBS mutant) revealed that a moderate number of genes affected were common (Fig. S8). However, a major number of genes affected in both cases did not belong to the same pathways. This indicates that along with affecting the Rho-dependent processes, the peptides could also have other physiological targets.

### Effect of peptides on the Rho proteins from pathogens

**Figure 9:**
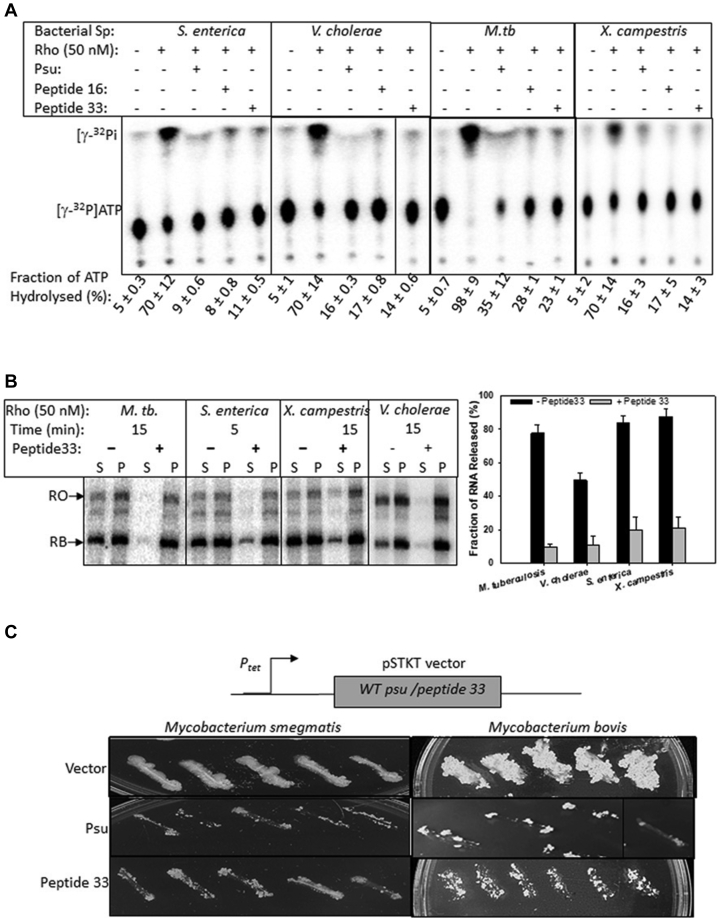
***In vitro* and *in vivo* effects of the peptides on the Rho proteins from some pathogens.***A*, inhibition of the ATPase activities of Rho proteins from different pathogenic bacteria by the peptides as indicated. The autoradiograms showing the fractions of ATP hydrolyzed by Rho in the presence or absence of 50 μM peptide 16 and peptide 33 and 5 μM Psu. Poly (rC) was used as a substrate in cases of *Mycobacteria* and *Xanthomonas* Rho, whereas RNA with the *λtR1* terminator was used for *Salmonella* and *Vibrio* Rho. SDs were obtained from two to three measurements. *B*, Rho-dependent RNA release assays from the roadblock complex (the same as described for Fig. 3*E*) in the absence and presence of 35 μM peptide 33. Fifty nanomolar of each of the Rho proteins from indicated pathogenic bacteria were used in these assays. The supernatant fraction (S) contains half of the released RNA, whereas the P fraction contains the rest of the sample (half of the supernatant + pellet). All the other experimental conditions were the same as in Figure 3*E*. The fraction of released RNA ([2S]/([S] + [P])) in each case is shown as bar diagrams. The SDs were calculated from two to three measurements. *C*, *Mycobacterium smegmatis* strain mc^2^155 was transformed with either a pSTKT empty vector or pSTKT plasmid expressing either peptide 33 or WT Psu. Transformants were subsequently streaked onto 7H10 plates. *Mycobacterium bovis* BCG strain was transformed with the same plasmids as above. Transformants of the *M. bovis* strain were obtained after 3 weeks, and colonies were further streaked to show the growth differences in the *M. bovis* strains upon expression of either peptide 33 or the WT Psu. The spliced part of the images in Figure 9, *A* and *C* are indicated by *vertical lines*.

Finally, we wanted to test whether these peptides can inhibit the functions of the Rho proteins of different pathogens. We have chosen the Rho proteins from *Xanthomonas campestris*, *Mycobacterium tuberculosis*, *Vibrio cholerae,* and *Salmonella enterica*. We first tested the inhibition of their respective RNA-dependent ATPase activities in the presence of Psu and peptides 16 and 33. We used poly (rC) as a cofactor to induce the ATPase function of the Mycobacterial and *Xanthomonas* Rho proteins, whereas RNA with the *λtR1* terminator was used in the cases of *Salmonella* and *Vibrio* Rho proteins. In an earlier study (13), we have observed that the rates of ATP hydrolysis of the *Salmonella* and *Vibrio* Rho proteins in the presence of poly(C) RNA are too fast to be inhibited by Psu (13). Hence, we used an RNA containing the *λtR1* terminator that elicits a slower rate of ATP hydrolysis. We observed that like Psu, both the peptides were able to inhibit the ATPase functions of the pathogenic Rho proteins very efficiently, even better than the *E. coli* Rho (compare Fig. 9*A* with Fig. 3*C*). It might be because the pathogenic Rho proteins are less efficient in utilizing the RNA substrates of *E. coli* Rho (polyC and *λtR1* terminator), and hence, peptides could inhibit their ATPase function on these RNA molecules more efficiently.

Next, we monitored Psu and peptide 33–induced effects on the RNA release by these Rho proteins from a stalled EC formed by the *E. coli* RNAP. The stalled ECs (RB) downstream of the *trpt*′ terminator was formed similarly as described in Figure 3*E*. Earlier, we have shown that the Rho proteins from the abovementioned pathogens are capable of releasing RNA from a stalled EC made of *E. coli* RNAP (13). We observed that like Psu, peptide 33 could inhibit the termination process by all the different Rho proteins (Fig. 9*B*). Therefore, the novel peptides are quite efficient in inhibiting the functions of the Rho proteins of different pathogens under *in vitro* conditions.

To be candidates for further development as antimicrobials, the peptides must cause lethality or toxicity in clinically relevant bacterial strains. We chose two of the *Mycobacterium* (gram positive) strains to study the *in vivo* effects of Psu and peptide 33. Because the Rho proteins of *M. tuberculosis* and *Mycobacterium bovis* have the same sequence, we monitored the *in vivo* effects in an *M. bovis* strain. We used *Mycobacterium smegmatis* mc^2^155 and *M. bovis BCG* to monitor the effect of *in vivo* expressions of Psu and peptide 33. We electroporated these two strains with pSTKT plasmids expressing either WT Psu (pRS1724) or peptide 33 (pRS1893) or with an empty vector (pRS1511). In this vector, the Psu gene and the peptide sequences are cloned under the control of an anhydrous tetracycline (ATc)-inducible *P*_*tet*_ promoter (Fig. 9*C*). The transformants obtained from the transformation of both the *mycobacterium* strains by pRS1724 (Psu) and pRS1893 (peptide 33) yielded fewer colonies than those obtained from the transformation with the empty vector, pRS1511, and upon streaking, exhibited a very sick phenotype (Fig. 9*C*). Interestingly, the abovementioned toxic effects of Psu and peptide 33 were observed in the absence of the inducer; hence, their basal level expressions were sufficient to induce lethality. Therefore, we concluded that like Psu, peptide 33 also functions as a strong inhibitor of *Mycobacterium* Rho *in vivo*.

## Discussion

The transcription terminator Rho is a highly conserved protein in bacteria and acts as a pleiotropic master regulator being involved in many physiological processes (4, 7). These features enable it to be a useful target for designing bactericidal agents. Here, we report the designing and characterization of novel anti-Rho peptides from C-terminal helix 7 of the bacteriophage P4 capsid protein, Psu, using phenotypic screening methods (Figs. 1 and S1*A*). We have chosen two 38-mer peptides, 16 and 33, for detailed analyses of their anti-Rho activities among the nine screened peptides. We have shown that upon expression, these two peptides induce lethality to the *E. coli* strains and inhibited *in vivo* transcription termination. The chemically synthesized peptides exhibited inhibition of *in vitro* transcription termination and RNA-dependent ATPase functions of Rho by directly interacting with the latter (Figure 2, Figure 3, Figure 4 and Fig. S4, *A* and *B*). The molecular modeling, dynamic simulations, and CD studies revealed that these two peptides are predominantly helical in their C-terminal region, and their N-terminal region is randomly coiled (Fig. 2 and Figs. 1*B*, S2, and S3). These two peptides could be further shortened by 8 to 10 amino acids from their C-terminal regions without affecting their functions significantly (Figs. 5, and S5*A*). The direct binding assays, molecular docking, and mutational and genetic analyses revealed that these two peptides bind near the primary RNA-binding region of the Rho hexamer at the interface of the two adjacent protomers, which is quite distinct from the Psu-binding sites (Figs. 6, 7 and Figs. S5, S6 and S7). However, the genome-wide effects on transcription of these peptides and Psu are largely indistinguishable (Figs. 8 and S8). These peptides were also able to inhibit functions of Rho from several pathogens and induced lethality to the *Mycobacterium* species (Fig. 9). Our results unequivocally established these novel 38-mer peptides as anti-Rho peptides and showed that they are capable of functioning as functional mimetic of the ∼42-kDa dimeric bacteriophage P4 capsid protein, Psu, from which they were derived. We concluded that these 38-mer peptides and their 8 to 10 amino acid deletion derivatives should provide ideal platforms for designing novel AMPs targeted to the conserved transcription terminator, Rho.

Over the last decade, it has been established that the Rho-dependent termination has a genome-wide effect (20, 21, 22), which directly or indirectly enabled Rho to function as a regulator of many physiological processes (4). Our laboratory has recently shown that Rho augments DNA repair (6) and controls bacterial antibiotic sensitivity *via* multipartite pathways (7). Likely, its RNA binding, as well as RNA translocase activities, could also impart its roles in various RNA metabolism processes, RNA chaperone activities, and RNA turnover functions. Although having such multifaceted *in vivo* roles, the Rho protein never elicited much traction among the researchers to establish it as a potent drug target for antibacterial treatment. To date, there is only one well-known antibiotic, bicyclomycin, that directly binds near the ATP-binding sites and inhibits Rho functions (23), which, however, has limited clinical use. The peptides that we have reported here define another class of Rho inhibitors that target the PBS of Rho. In this regard, these peptides functionally resemble the other two PBS-binding Rho antagonists, YaeO and Hfq (24, 25, 26). Hence, both the ATP-binding pocket in the central channel and the N-terminal RNA-binding sites offer target areas for designing novel Rho inhibitors.

Many biologically active peptides have been produced from the functional domains of the antimicrobial proteins of higher eukaryotes that are components of their innate immune system, such as lactophoricin, lactoferrin (27), lysozyme (28, 29), and so forth. In a different approach, synthetic AMPs were also synthesized from the naturally occurring antimicrobials. AWRK6 is a synthetic peptide that was derived from the frog-skin antimicrobial Dybowskin-2CDYa (30), several potent AMPs were produced from the plant antimicrobial protein LsGRP1 of *Lilium* (31) and the synthetic peptide In-58 was derived from indolicidin (32). Functional peptides were also designed based on the structures of HIV-1 protein gp120, chemotaxis inhibitory protein of *Staphylococcus aureus* (33, 34, 35), and so forth. To our knowledge, this is the first report where the directed evolution method comprising random mutagenesis of a region of a bacteriophage protein was used to design novel peptides having anti-Rho peptides. Bacteriophages are the reservoirs of thousands of genes coding for proteins that interact and modulate host machinery. Our methodology opens up the possibilities of designing many different types of peptides from the bacteriophage proteins functional against the bacterial machinery, which in turn could be used for designing AMPs.

These 38-mer peptides are too large to be delivered into any bacteria. Sequential deletion from the C-terminal region keeping the core amino acids intact revealed that the peptides could be shortened by ten more amino acids, which is still not small enough for its delivery. It will be required to further mutagenize the core amino acids together with shortening their length. Alternatively, the plasmid DNA containing the nucleotide sequences of the peptides could be delivered to the bacteria *via* conjugation methods or by exploiting the properties of natural transformation competence of several pathogenic bacteria or by delivering the desired DNA sequences using engineered bacteriophages.

## Experimental procedures

### Materials

NTPs were purchased from GE Healthcare. [γ-^32^P] ATP (3000 Ci/mmol) and [α-^32^P] CTP (3000 Ci/mmol) were obtained from Jonaki, Board of Radiation & Isotope Technology (BRIT, Hyderabad, India), or American Radiolabeled Chemicals, Inc (ARC). Antibiotics, IPTG, lysozyme, DTT, and bovine serum albumin (BSA) were obtained from United States Biochemical Corporation (USB). Restriction endonucleases, WT *E. coli* RNA polymerase holoenzyme, and T4 DNA ligase were obtained from New England Biolabs (NEB). Streptavidin-coated magnetic beads were purchased from Promega. Taq DNA polymerase was obtained from Roche Applied Science. Ni-NTA agarose beads were from Qiagen and Sigma. Peptides 33 and 16 of >98% purity were synthesized by Biotech Desk Pvt. Ltd (Hyderabad, India). Among different peptides tested, the His-tag version of these two peptides was only water soluble, and hence, we performed *in vitro* experiments with them.

Details of the bacterial strains, plasmids, and oligos are described in Tables 1 and S2.

**Table 1:** Bacterial strains and plasmids used in this study

Strains	Genotype	Reference
BL21(DE3)	*F-omp ThsdSB*(rB-mB-) *gal* dcm(DE3)	Novagen
DH5α	Δ (*argF-lac*) U169 *supE*44 *hsdR17 recA1 endA1 gyrA96 thi-1 relA1*(ø80lacZΔM15)	Novagen
GJ3161 (RS257)	MC4100 *galEp3*	(52)
XL1-Red	*endA1 gyrA96 thi-1 hsdR17 supE44 relA1 lac mutD5 mutS mutT Tn10Tet*	Stratagene
RS734	MC4100, λRS45 lysogen carrying P_lac_-*tR1-lacZYA*	
RS659	MG1655 *Δrho:: Kan* with pHYD1201	(16)
RS1263	*E. coli* MG1655; K-12; WT	Lab stock
RS1487	*M. smegmatis* MC^2^155; WT	Dr. Sangita Mukhopadhyay
RS1838	*M. bovis* BCG; WT	Dr. Sanjeev Khosla
RS2045	MG1655, *Δrac*, *Δlac*, λRS45 lysogen carrying P_lac_-*trpt*′*-lacZYA*	This study
RS2046	MG1655, *Δrac*, *Δlac*, λRS45 lysogen carrying P_lac_-*t*_*rac*_*-lacZYA*	This study
RS2047	MG1655, *Δrac*, *Δlac*, λRS45 lysogen carrying P_lac_-*tR1-lacZYA*	This study

Plasmids	Description	Reference
pRS96	pET21b with *E. coli rho* cloned at NdeI/XhoI site, encoding His tag at C-terminal domain; Amp^R^	(16)
pRS106	pT7A1 clone at EcoRI/HindIII sites upstream of *trpt*′ cloned at HindIII/BamHI sites of pK8641; Amp^R^	(9)
pRS258	pNL150 with P_tac_-WT *psu*; Cam^R^	(53)
pRS458	pET28b with WT *psu* cloned at NdeI/XhoI site, encoding His tag at N-terminal domain; Kan^R^	(9)
pRS553	pET28a with *M. tuberculosis rho* cloned at NdeI/XhoI site, encoding His tag at N-terminal domain; Amp^R^	(54)
pRS1511	pSTKT *mycobacterial* shuttle vector with P_tetO_; Kan^R^	(55)
pRS1672	pET28b with *Xanthomonas campestris rho* cloned at NdeI/XhoI site, encoding His tag at N-terminal domain; Kan^R^	(13)
pRS1680	pET28b with *Salmonella enterica rho* cloned at NdeI/XhoI site, encoding His tag at N-terminal domain; Kan^R^	(13)
pRS1724	pSTKT with WT *psu* cloned at EcoRI/HindIII site; Kan^R^	(13)
pRS1770	pET28a with *Vibrio cholerae rho* cloned at NdeI/BamHI site, encoding His tag at N-terminal domain; Kan^R^	Dr. U. Sen
pRS1888	pNL150 with Peptide 16 (as obtained in screening); Cam^R^	This study
pRS1889	pNL150 with Peptide 33 (as obtained in screening); Cam^R^	This study
pRS1893	pSTKT with Peptide 33; Kan^R^	This study
pRS1894	WT α-7 peptide with adjacent vector sequence in pLN150; Cam^R^	This study
pRS1895	16 Peptide without adjacent vector sequence in pNL150; Cam^R^	This study
pRS1896	33 Peptide without adjacent vector sequence in pNL150; Cam^R^	This study
pRS1958	His 33 peptide Δ4 CTD in pNL150; Cam^R^	This study
RS1959	His 33 peptide Δ6 CTD in PNL150; Cam^R^	This study
RS1960	His 33 peptide Δ8 CTD in PNL150; Cam^R^	This study
pRS2032	pNL150 with 2X His peptide 33 cloned at EcoRI/HindIII; Cam^R^	This study
pRS2033	pNL150 with 3X His peptide 33 cloned at EcoRI/HindIII; Cam^R^	This study
pRS2034	pNL150 with 4X His peptide 33 cloned at EcoRI/HindIII; Cam^R^	This study
pRS2035	pNL150 with 2X His peptide 33 Δ8 cloned at EcoRI/HindIII; Cam^R^	This study
pRS2036	pNL150 with 3X His peptide 33 Δ8 cloned at EcoRI/HindIII; Cam^R^	This study
pRS2037	pNL150 with 4X His peptide 33 Δ8 cloned at EcoRI/HindIII; Cam^R^	This study
pRS2048	pNL150 with peptide 33 Δ10 CTD cloned at EcoRI/HindIII; Cam^R^	This study
pRS2049	pNL150 with peptide 33 Δ12 CTD cloned at EcoRI/HindIII; Cam^R^	This study

### Preparation of randomly mutagenized library of Psu–C-terminal helices to screen for peptides

First, we identified important amino acids of helix 7 of Psu involve in interacting with Rho from the structural and cross-linking data (11), such as N174, F177, S181, and L184. We randomly mutagenized these positions by using oligos carrying random bases at the first and second bases individually and at both the bases of each of the codons (Fig. 1*A*). Random mutations in these positions were introduced by PCR. The library of PCR products thus obtained were then cloned into an *in vivo* expression vector pNL150 under the control of an IPTG-inducible *P*_*tac*_ promoter. The cloning was done at the *EcoRI/HindIII* sites of the pNL150 vector.

### Screening of the gain-of-function peptides

For isolation of the gain-of-function mutant peptides from the abovementioned library, the library was transformed into the strain RS734 that contains a chromosomal copy of a reporter cassette downstream of a Rho-dependent terminator (*P*_*lac*_*-H19BnutR/tR1-lacZYA*). The transformants were selected on LB–X-gal plates containing 20 μM IPTG. The colonies on this indicator plate would appear blue, only if the expressed peptides inhibit Rho function inducing antitermination at the *tR1* terminator of the reporter cassette. The blue colonies were purified by repeated streaking and were checked in the growth assays. The blue colonies that were defective for growth in the presence of high IPTG concentrations (>100 μM) were selected. Plasmids expressing peptides from these selected strains were then isolated, and the peptide sequences were confirmed by sequencing. About ∼80,000 colonies were screened in the process.

### Growth assays

The *E. coli* MG1655 strain (RS1263) was transformed with the pNL150 vector expressing either full-length Psu or the peptides derived from the Psu CTD from an IPTG-controlled *P*_*tac*_ promoter. The empty vector was also transformed as a negative control. After the transformation, the growth assays of the transformants were performed in the LB media supplemented with different concentrations of IPTG in a 96-well microtiter plate. The growth kinetics was recorded in a SpectraMax M5 microtiter plate reader at 37 °C for 6 h. To measure the growth kinetics of these clones in the presence of different Rho mutants (R144E, R146E, and E148R; 11), RS659 (MG1655 Δ*rho:: kan*; IPTG-dependent shelter plasmid pHYD1201 expressing WT Rho) was at first transformed with pCL1920 expressing WT and the Rho mutants. After removal of the shelter plasmid, these strains were transformed with pNL150 expressing either WT Psu and or the peptides, and the growth assays were performed as before. Different deletion mutants of peptides 33 and 16 were prepared on the pNL150 vector either by the PCR amplification of the desired region followed by cloning or by site-directed mutagenesis, and growth assays were performed using the same strain and method used for the cases of the full-length peptides.

### *In vivo* Rho-dependent termination assays

We used two *lacZ* reporter systems for *in vivo* Rho-dependent transcription termination assays. They are *P*_*lac*_*-trpt*′*-lacZAY* (RS2045) and *P*_*lac*_*-t*_*rac*_*-lacZAY* (RS2046), where the terminators, *trpt*′ and *t*_*rac*_, were fused upstream of the *lacZ* genes, and the transcription in all the reporters were directed from the *P*_*lac*_ promoter. These reporters are inserted in the chromosome of *E. coli* MG1655 *Δrac Δlac* strain by λRS45 transduction. These reporter strains were transformed with pNL150 plasmid expressing peptide 33 and Psu and also with the empty vector. The transformants were streaked on LB–X-gal plates containing different concentrations (0 μM, 25 μM, 50 μM, 100 μM, and 250 μM) of IPTG and incubated at 37 °C until the blue color was developed. Whenever required, we also used the *galEP3* reporter system for the *in vivo* Rho-dependent termination assay. In this reporter, a series of terminators were inserted in the form of the IS2 element at the beginning of the galactose operon. The strain RS257, containing the galEP3 reporter cassette, was transformed with the pNL150 plasmid expressing WT Psu, peptides 16 and 33, and also the empty vector. The resulting strains were streaked on MacConkey agar plates supplemented with 1% (w/v) galactose and incubated at 37 °C till pink/red colors were developed.

For the qRT-PCR assays, strains RS2045 (λRS45 lysogen carrying *P*_*lac*_-*trpt*′*-lacZYA*) and RS2046 (λRS45 lysogen carrying *P*_*lac*_-*t*_*rac*_-*lacZYA*) were transformed with pNL150 plasmid expressing peptide 16, peptide 33, peptide 33Δ8, peptide 33Δ10, Psu, and also with the empty vector. After induction with 100 μM IPTG (20 μM IPTG for Psu and 50 uM for peptide33Δ8), cells were harvested in the log phase (absorbance at 600 nm ∼0.3–0.4). The total RNA was isolated using the RNeasy Plus Mini Kit (Ambion), and the cDNA was made using random hexamers and SuperScript III Reverse Transcriptase. The amount of lacZ expression was checked by qRT-PCR using the primers RS1993 and RS1994. The amount of cDNA produced during the PCR cycles was monitored in real time using SYBR green dye in the Applied Biosystems 7500 RT-PCR system. The threshold cycle C_t_ was calculated from the midpoint of the sigmoidal curve obtained by plotting the fluorescence intensity against the number of PCR cycles. The fold change was calculated using 2−^ΔΔCt^ in the mRNA level in the presence of the peptides or Psu, with respect to the empty vector, where C_t_ = the number of threshold cycle; ΔC_t_ = (C_t_ of target gene – C_t_ of internal control); and ΔΔC_t_ = ΔC_t_ in presence of peptides or Psu –ΔC_t_ in the presence of an empty vector. The level of *rpoC* mRNA was used as an internal control. We have earlier observed that unlike the ribosomal RNAs, the *rpoC* mRNA level does not change upon inhibition of the Rho function (19). Hence, we used the level of this *rpoC* RNA as an internal control. The primer pairs of 200-nt size were designed corresponding to the middle region of the test genes. The C_t_ values obtained from all the qRT-PCR experiments (Figs. 3*B* and 8*D*) are described in Table S1.

### CD spectrometry

CD spectra of peptides 16 and 33 dissolved in 10 mM 2-(N-morpholino)ethanesulfonic acid at pH 5.5 were recorded using a Jasco J-810 spectrometer (Jasco Spectroscopic Company). The peptides are properly soluble at a mildly acidic pH, so a 2-(N-morpholino)ethanesulfonic acid buffer at pH 5.5 was used. The CD spectra from 190 to 270 nm wavelengths were recorded at 25 °C. The estimation of secondary structure was done according to a published method using the manufacturer's software for the instrument (36). The molar ellipticity was calculated based on the concentrations of the peptides used.

### In silico modeling and simulation of the peptide structure

The 3D structure of peptides 16 and 33 was predicted using Iterative Threading ASSEmbly Refinement server (37, 38, 39). The server reported five models, among which the model with the highest confidential score was subjected to MD simulation, using GROMACS-2018 to obtain the lowest energy conformation (40). CHARMM 36 all-atom force field parameters were applied, and the peptide was placed at the geometrical center of a triclinic box with a minimum 1.0 nm distance from the edges of the box (41). This box was then solvated with simple point-charge water molecules, and the charge of the system was neutralized with the counterions. The entire system was then energy minimized with the steepest descent algorithm and equilibrated with NVT (constant Number of particles, Volume, and Temperature) and NPT (constant Number of particles, Pressure, and Temperature) ensembles. Finally, the MD run of both peptides was performed in the constant Number of particles, Pressure, and Temperature ensemble for a time scale of 1 μs. The principal component analysis and the free-energy landscape were integrated with structural clustering analysis to identify the representative structure of the peptides (42). The structure validation was performed by PROCHECK Ramachandran plot using the Structure Analysis and Verification server (http://servicesn.mbi.ucla.edu/PROCHECK/; (43)). The same MD run protocol was used for the protein–peptide complex for a 200-ns time scale.

### Molecular docking of peptides onto the 3D structure of the Rho hexamer

Before the molecular docking, we modeled the missing residues in Rho (closed) structure by Modeller 9v20 using the crystal structure of Rho (3ICE) as a template (44, 45). The ATP mimic (ADP.BeF_3_) present in the crystal structure was replaced by ATP. The binding region(s) of His peptide 33 and non–His peptide 33 on Rho (close) structure was predicted by protein–peptide docking in the ClusPro web server (46). After the successful docking, the hot spot residues (a residue or a subset of residues that contributes most to the binding affinity) that forms the protein–protein/peptide interface were identified using the online web services KFC server (http://kfc.mitchell-lab.org/) and HotRegion database (http://prism.ccbb.ku.edu.tr/hotregion/; (47, 48)).

### ATPase activities of Rho

The RNA-dependent ATP hydrolysis of Rho in the absence and presence of the peptides or WT Psu were measured using RNA containing the *λt*_*R1*_ terminator sequence. ATP hydrolysis was assayed by monitoring the release of Pi from [γ-P^32^] ATP (3500 Ci/mmol; BRIT, India) as was observed on polyethyleneimine TLC plates essentially following the methods described earlier (16) in T buffer (25 mM Tris HCl [pH 8.0], 50 mM KCl, 5 mM MgCl_2_, 1 mM DTT, and 0.1 mg/ml BSA) at 37 °C. The ATP hydrolysis of 1 mM ATP mixed with [γ-^32^P] ATP was measured using 50 nM Rho. The reactions were initiated by the addition of 200 nM RNA. The release of Pi was analyzed by exposing the TLC sheets to a PhosphorImager screen and subsequently by scanning using an FLA 9000 system (Typhoon). To examine the Rho inhibition, assays were performed in the presence or absence of peptide 16, peptide 33, and WT Psu and were stopped at a final time point where maximum hydrolysis occurred. For the *X. campestris* and *Mycobacterium tuberculosis* Rho proteins, poly(rC) was used as the template, as these Rho proteins could efficiently be inhibited by the peptides or Psu on this RNA, unlike the *E. coli* Rho. In the case of the Rho proteins from *Salmonella typhimurium* and *Vibrio cholera*, *λt*_*R1*_ RNA was used as the template. The rates of ATPase activities of Rho both in the presence and absence of peptides (Fig. 7*C*) were measured from the plots of the fraction of ATP that remained against time. The exponential decay of the form exp(-λx) was used to calculate the rate (λ) of the reactions.

### *In vitro* transcription and RNA release assays

*In vitro* Rho-dependent transcription termination assays were performed in transcription buffer (T-buffer; 25 mM Tris HCl, pH 8.0, 50 mM KCl, 5 mM MgCl_2_, 1 mM DTT, and 0.1 mg/ml BSA) at 37 °C. The plasmid pRS106 containing the strong Rho-dependent terminator, *trpt*′, fused downstream of the T7A1 promoter was used to amplify a linear DNA template having this T7A1-*trpt*′ cassette. The reactions were initiated with 10 nM DNA template, 50 nM WT RNAP, 175 μM ApU, 5 μM GTP and 5 μM ATP, and 2.5 μM CTP to make a 23-mer EC_23_. [α-32P] CTP (3000 Ci/mmol; BRIT, India) was added to label the transcripts. The EC_23_ was then chased with 25 μM NTPs in the presence of 10 μg/ml rifampicin for 15 min at 37 °C in either the absence or presence of 50 nM Rho. To observe the effects of peptide 33, different concentrations of peptides were added to the chase solution. The reactions were stopped by phenol extraction followed by ethanol precipitation. Reaction products were then separated using 8% sequencing gel, and the image was taken in phosphorimager. The amount of the run-off product was calculated using Image Quant 5.2.

For the RNA release assays, the linear DNA template was PCR amplified from the plasmid pRS106 (*P*_*T7A1*_*-trpt*′) using the RS83/RS177 oligonucleotide pairs. To form a stalled EC using a lac repressor RB on the template, a 22-bp *lac* operator sequence was inserted after the *trpt*′ terminator sequence using a downstream primer (RS177) carrying the operator sequence (49). To immobilize the DNA templates onto the streptavidin-coated magnetic beads (Promega), a biotin group at the 5′ end of the templates was incorporated by using a biotinylated primer (RS83). Transcription was performed on this *P*_*T7A1*_*-trpt*′*-lacO* template immobilized on streptavidin-coated magnetic beads in the presence of rifampin in the T-buffer (25 mM Tris-HCl [pH 8.0], 5 mM MgCl_2_, 50 mM KCl, 1 mM DTT, and 0.1 mg/ml BSA). A 100 nM concentration of the lac repressor was added to the DNA templates to form the RB. On this template, EC_23_ was formed in the same way as described above. This complex was then chased with 250 μM of each of the NTPs. After the chase, excess NTPs were removed by washing the beads thoroughly with T buffer. Rho (50 nM), peptide 16 (50 μM), peptide 33 (35 μM), and Psu (5 μM) were added to each of the reactions as indicated in the figures, and 10 μl of samples was removed after 10 min and was separated into S (half of the supernatant) and S + P (the other half of the supernatant plus the pellet) by keeping the microfuge tubes held against a magnetic stand. Samples were run on an 8% sequencing gel and were analyzed by an FLA 9000 PhosphorImager (Typhoon). The fractions of released RNA, {2S/[S + (S + P)]}, were measured from the band intensities.

In our earlier study, the Rho, as well as the Psu functions, were found to be optimal in the T-buffer (9). Hence, we conducted both the ATPase and the transcription termination assays in T-buffer and followed similar protocols as described earlier (9).

### *In vitro* pull-down assay

For *in vitro* pull-down assays, 30 μg of the His-tagged peptide 16, 20 μg of the His-tagged peptide 33, 15 μg of His-tagged WT Psu, and 5 μg of the non–His-tagged Rho protein (P167L Rho) were used. The His-tagged peptides and Psu were first bound to 100 μl of pre-equilibrated Ni-NTA beads and incubated at 4 °C for 12 to 16 h in the binding buffer (100 mM NaH_2_PO_4_, pH 8.0; 100 mM NaCl, 10 mM imidazole, and 1 mM PMSF). Non–-His-tagged Rho was then added to this ensemble in the presence of 1 mM ATP and incubated at 37 °C for 20 min. The supernatant was removed after spinning the mixture at 2000 rpm for 2 min. The beads were then washed with 100 μl of wash buffer (100 mM NaH_2_PO_4_, 100 mM NaCl, and 50 mM imidazole), and the proteins were eluted with 100 μl of the elution buffer composed of 100 mM NaH_2_PO_4_, 100 mM NaCl, and 500 mM imidazole. Samples were electrophoresed on 18% SDS-Tricine PAGE. The pull-down assays were performed in the phosphate buffer as the binding on Ni-NTA beads is optimum under this condition.

### ITC for Rho–peptide binding

Binding characteristics of peptide 33 and peptide 16 with Rho P167L protein were examined with ITC using a Nano-ITC Low-Volume isothermal titration calorimeter (TA Instruments). 1 μM Rho P167L in the ITC buffer (25 mM Tris Cl, pH 8.0, 50 mM KCl, 100 mM NaCl, 5 mM MgCl_2_, 1 mM ATP, 0.01% Triton X-100, and 5% glycerol) was titrated against 50 μM peptide solution prepared in the same buffer. ITC experiments were performed at 25 °C by injecting 2.5-μl injections of peptide solution into the Rho solution with constant stirring at 200 rpm. To identify the probable binding region(s) of peptide 33 on the Rho protein, ITC was performed in the presence of 10 μM Poly(dC)_34_, 2 mM Poly(rC), or 5 μM NusG-CTD premixed with the Rho protein under the same conditions described above. The thermodynamic parameters of all the interactions were obtained using the in-built software of the calorimeter. The specific buffer composition in these experiments was chosen to minimize the heat of dilution and noise and to reduce aggregation during a slow stirring inside the ITC cells used during the injections of the peptides.

### Microarray analyses

The MG1655 strain was at first transformed with pNL150 plasmids expressing WT Psu, peptide 33, peptide 33CTDΔ8, and the empty vector. Overnight cultures of these strains were subcultured in 10 ml LB with appropriate antibiotics and were allowed to grow until absorbance at 600 nm ∼0.3 to 0.4. Cultures were then spun down, and the cell pellet was resuspended in 1 ml of RNAlater . RNA isolation and the microarray experiments were performed by Genotypic Technology, Bangalore, India. The methods have been described in detail earlier (50). Two independent biological replicates for each strain were taken for the analyses. A fold change in gene expression for each strain was calculated with respect to the empty vector control. We have considered the genes whose expressions were changed 5-fold (log 2) or more with respect to the empty vector control.

### *In vivo* growth inhibition assays in *Mycobacterium*

Peptide 16, peptide 33, and WT Psu sequences were PCR-amplified from pNL150 containing these sequences by Deep Vent DNA polymerase (NEB) and were cloned into the *EcoRI/HindIII* sites of the plasmid pSTKT, a mycobacterial/*E. coli* shuttle vector carrying an ATc-inducible *P*_*tetO*_ promoter. Next, we electroporated the clones carrying the peptides, the WT Psu, and the empty vector into the *M. smegmatis mc*^*2*^*155* strain according to the published protocol (51). Transformants were grown at 37 °C for 4 days in Difco Middlebrook 7H10 agar plates supplemented with 10% oleic acid–albumin–dextrose–catalase and 0.2% glycerol in the presence of 10 μg/ml kanamycin and were further purified on a higher concentration (25 μg/ml) of a kanamycin-containing medium. Subsequently, colonies were patched onto agar plates supplemented with ATc to study the effect of the expressions of the WT Psu and the peptides on the viability of *M. smegmatis* cells. In the same way, the *M. bovis* strain was transformed with plasmids carrying Psu and peptide clones by following the procedures described above. In this case, the transformants were grown for 3 weeks under the same conditions as used for *M. smegmatis*. The transformants thus obtained were subsequently restreaked to assess the growth.

## Data availability

All the final data in the form of graphs and plots are available in the article. The corresponding raw data will be available upon request from the corresponding author, Ranjan Sen, CDFD, Hyderabad; email: rsen@cdfd.org.in.

## Supporting information

This article contains supporting information.

## Conflict of interests

R. S. is a DBT (Department of Biotechnology) -TATA innovation fellow. G. G. was a UGC (University Grant Commission) Senior Research Fellow. P. V. S. is a CSIR (Council for Scientific and Industrial Research) Junior Research Fellow. An Indian patent application (Application No. 201841048582) has been submitted for the designed peptides. The other authors declare that they have no conflicts of interest with the contents of this article.
