# Supplementary material for: Design of novel peptide inhibitors against the conserved bacterial transcription terminator, Rho
Source: J Biol Chem. 2021 May 15;296:100653. doi: 10.1016/j.jbc.2021.100653 (PMC8141534; doi:10.1016/j.jbc.2021.100653)
Supplement: Figures S1 to S8 and Tables S1 and S2 [file mmc1.pdf]

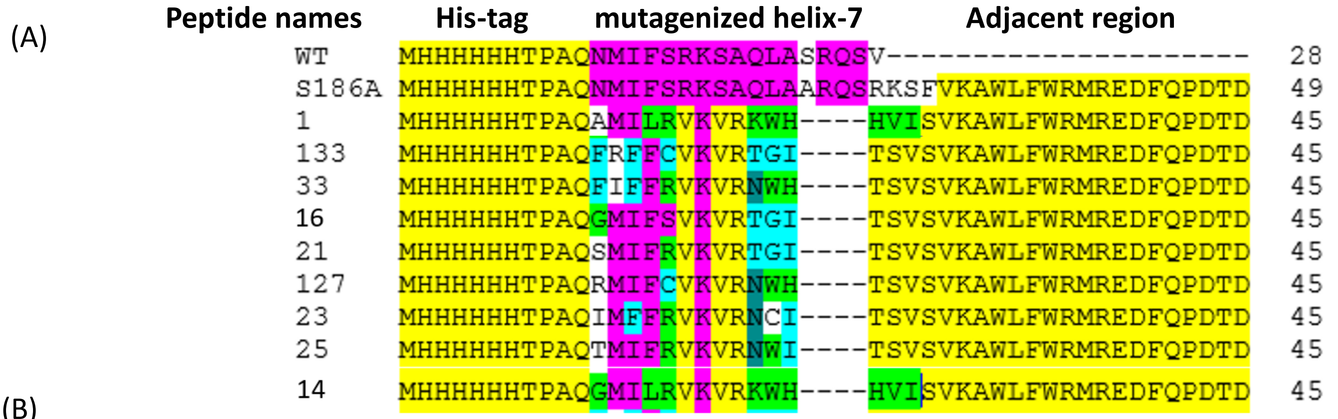

(B)
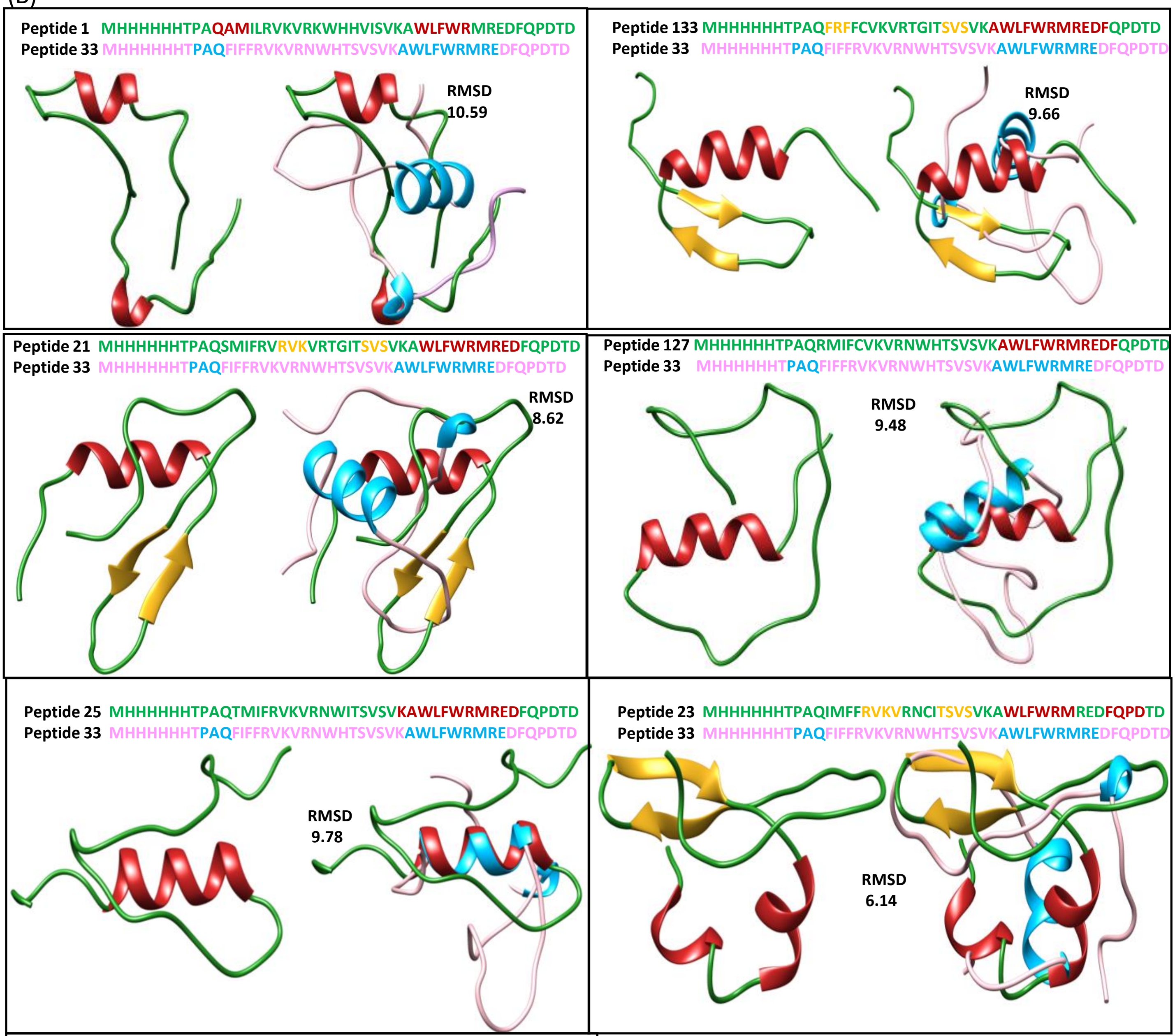

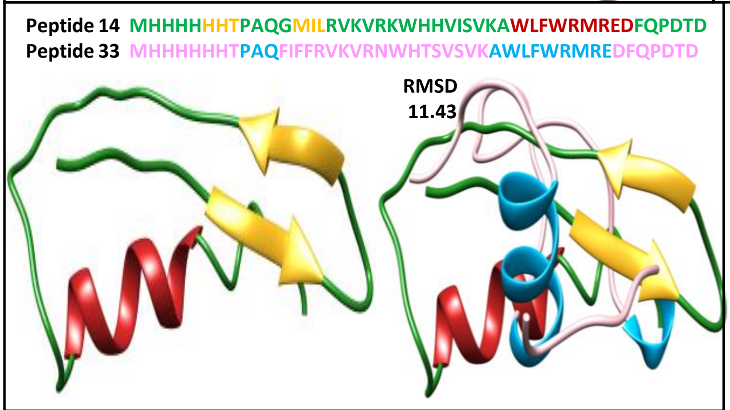

Figure S1: (**A**) Aligned sequences of all the different peptides obtained from the genetic screening for anti-Rho peptides. (**B**) Model structures of the indicated peptides after 1 μs of molecular dynamics (MD) simulation and their overlapped structure with peptide 33, shown next to each structures. The conformation of each of these peptides has RMS deviation (RMSD) in the range of 6.14 Å to 11.43 Å. The MD simulations were performed in a similar way as in figure S2.

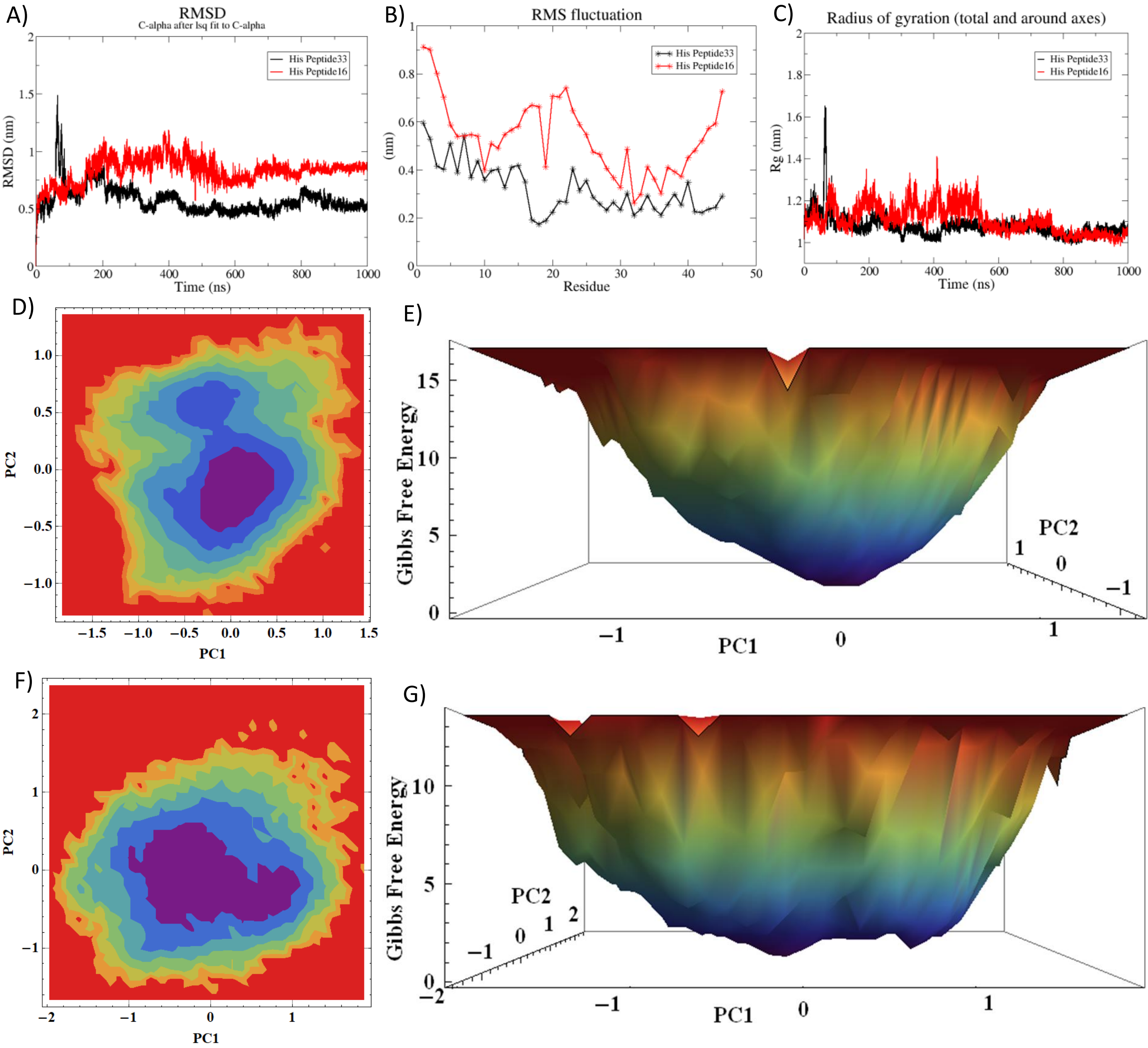

Figure S2: Molecular dynamics simulations of the peptides: (**A**) Root mean square deviation (RMSD) plots of peptide 33 (black) and peptide 16 (red) show that the conformations of both the peptides stabilized during a 1 μs simulation. The former one stabilized at an average of ~0.5 μs and the later at ~0.85 μs. (**B**) Residue-based root mean square (RMS) fluctuations of the peptide residues over the simulation time period. RMS fluctuations indicate that the displacement of each residues from its mean position. Except the 17-20 residues, the overall RMS fluctuation of peptide 33 is lower than that of peptide 16. (**C**) The fluctuations in the radius of gyration (Rg) is shown. The decrease in Rg values towards the end of the simulation time period indicates that the peptide structures have attained a stable folding. (**D**) The 2D and (**E**) 3D contour maps of peptide 33 obtained from free energy landscape (FEL) analysis, while (**F**) and (**G**) are for peptide 16. These maps elucidated single minimum energy cluster from which the lowest energy representative structure was extracted as shown in figure 2.

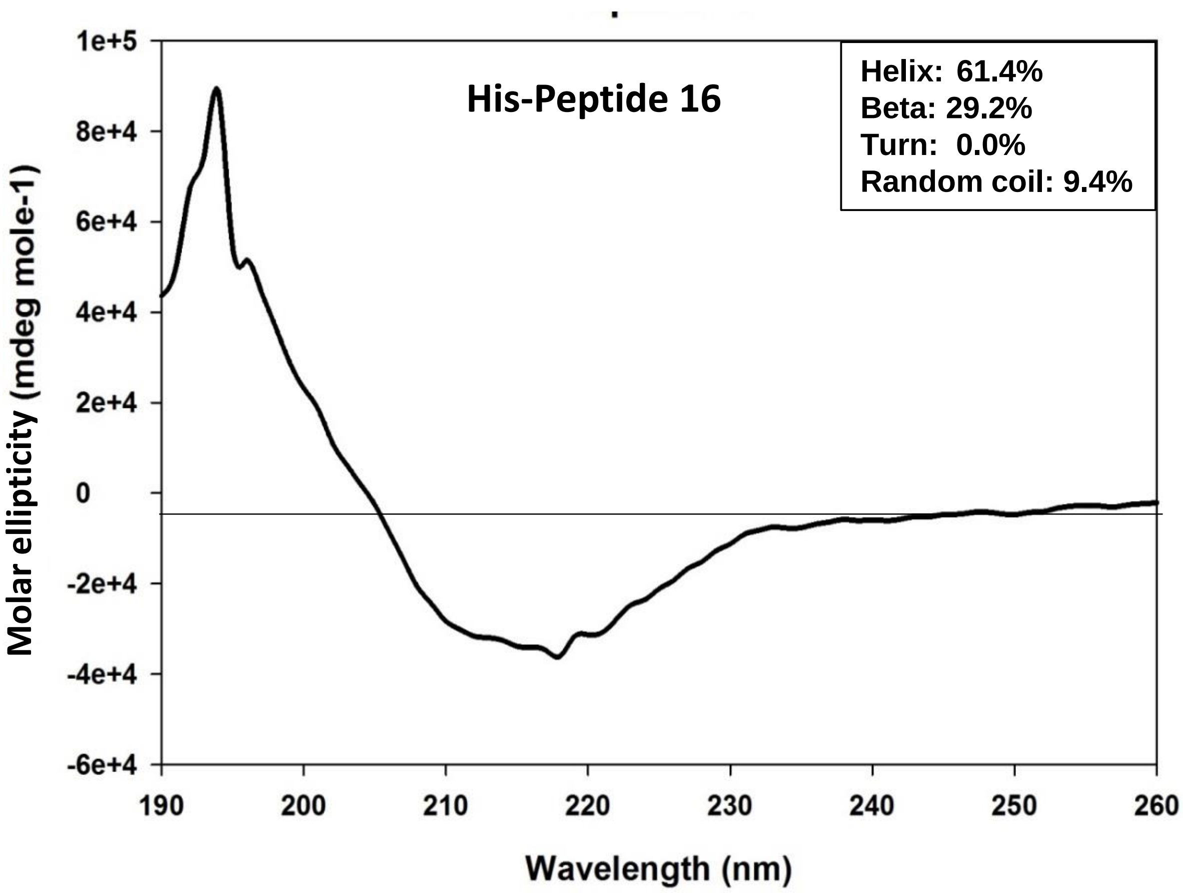

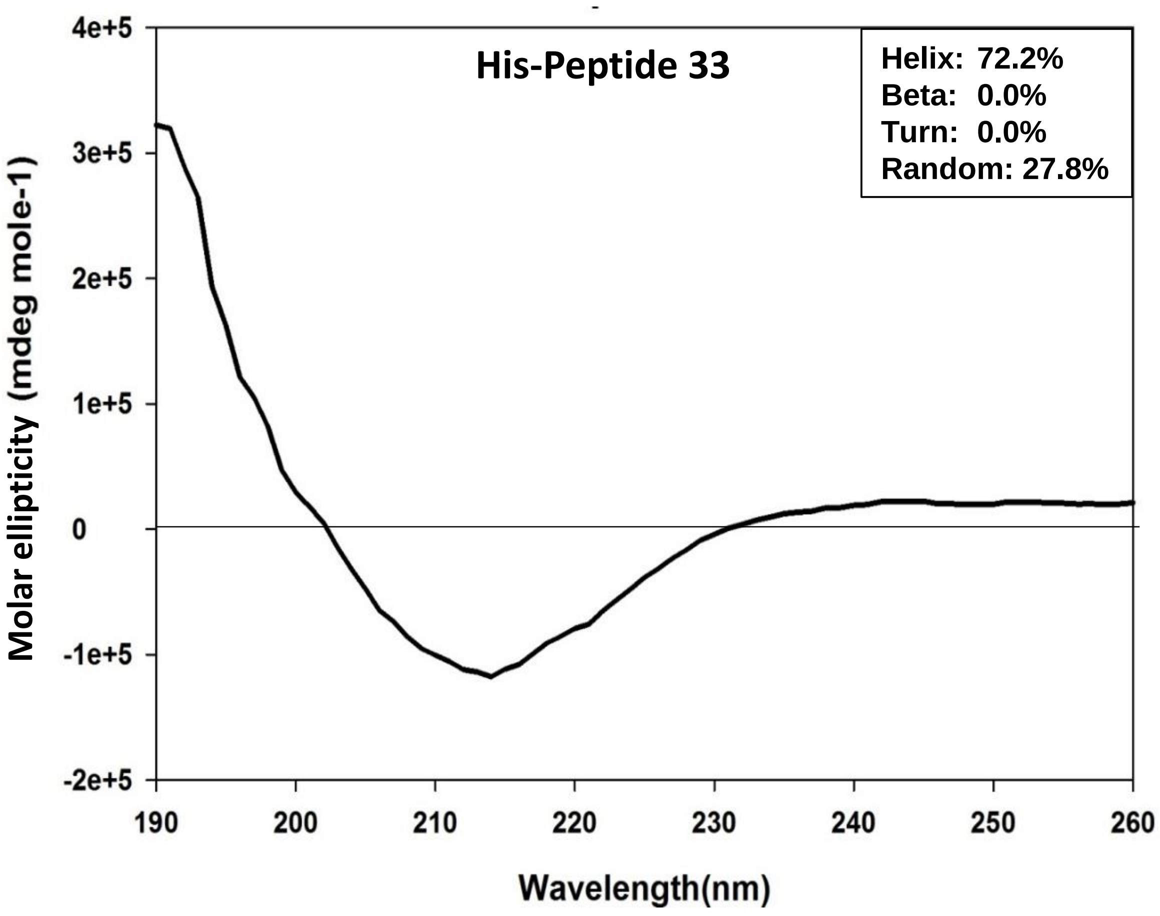

Figure S3: Circular Dichroism spectra of peptide 33 and 16 as indicated. Secondary structure estimations are also shown in the plots.

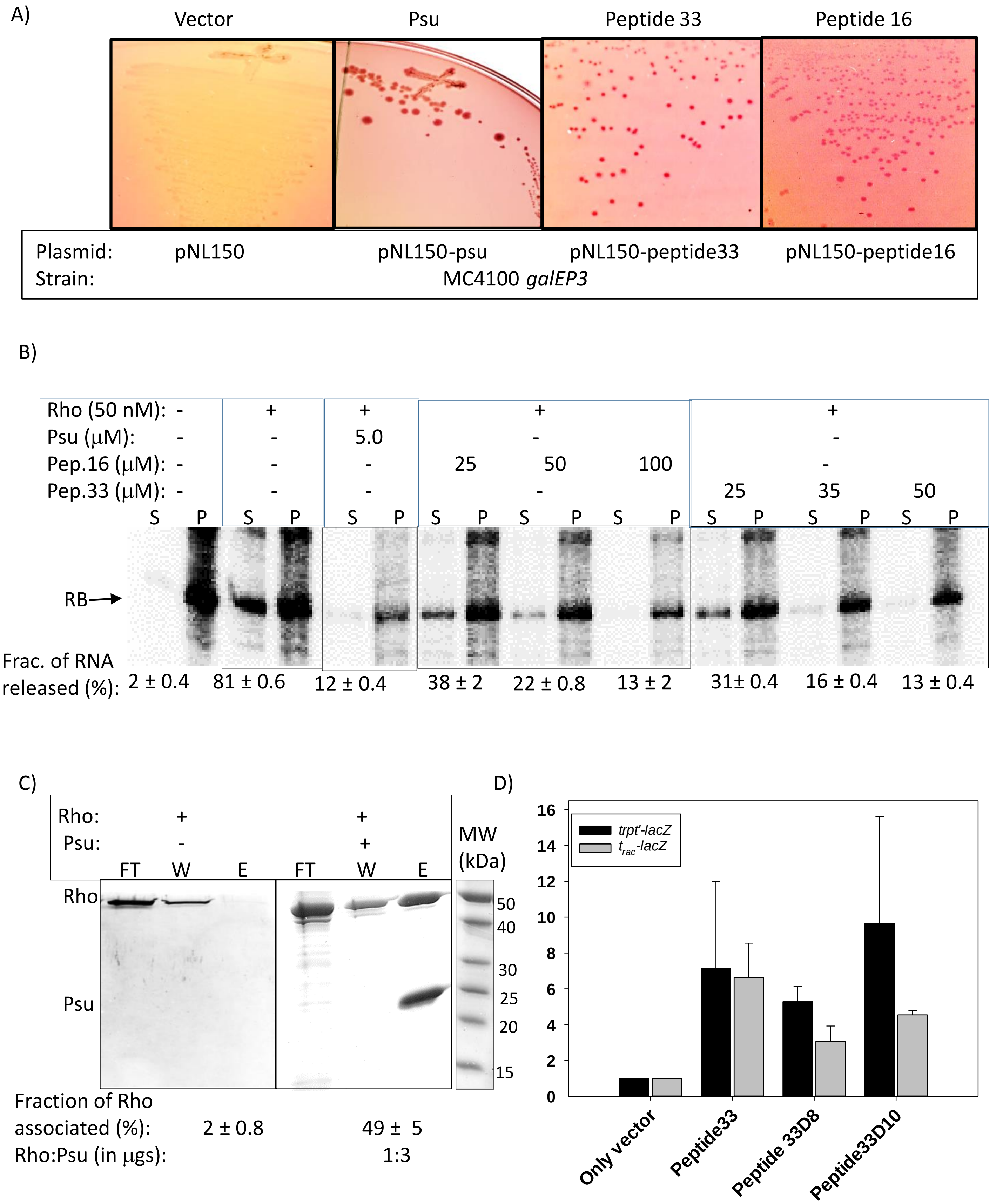

Figure S4: **A)** Colour-development in the MacConkey-galactose plates upon streaking of the indicated strains. **B**) Inhibition of *in vitro* RNA release by Rho from the Road-block complexes (RB) in the presence Psu and the peptides as indicated. Experiments were done in a same way as in figure 3D. Standard deviations are indicated. **C**) Fraction of Psu associated with P167L Rho. Experiments were performed in the same way as in figure 4A. Standard deviations are indicated. Aligned molecular weight markers are shown. **D)** Expression of *lacZ* measured by qRT-PCR expressed as fold change in Ct values *w.r.t* the empty vector. Indicated peptides are expressed in the strains described in figure 3A and experiments were done in a same way as in figure 3B. Error bars were obtained from standard deviations.

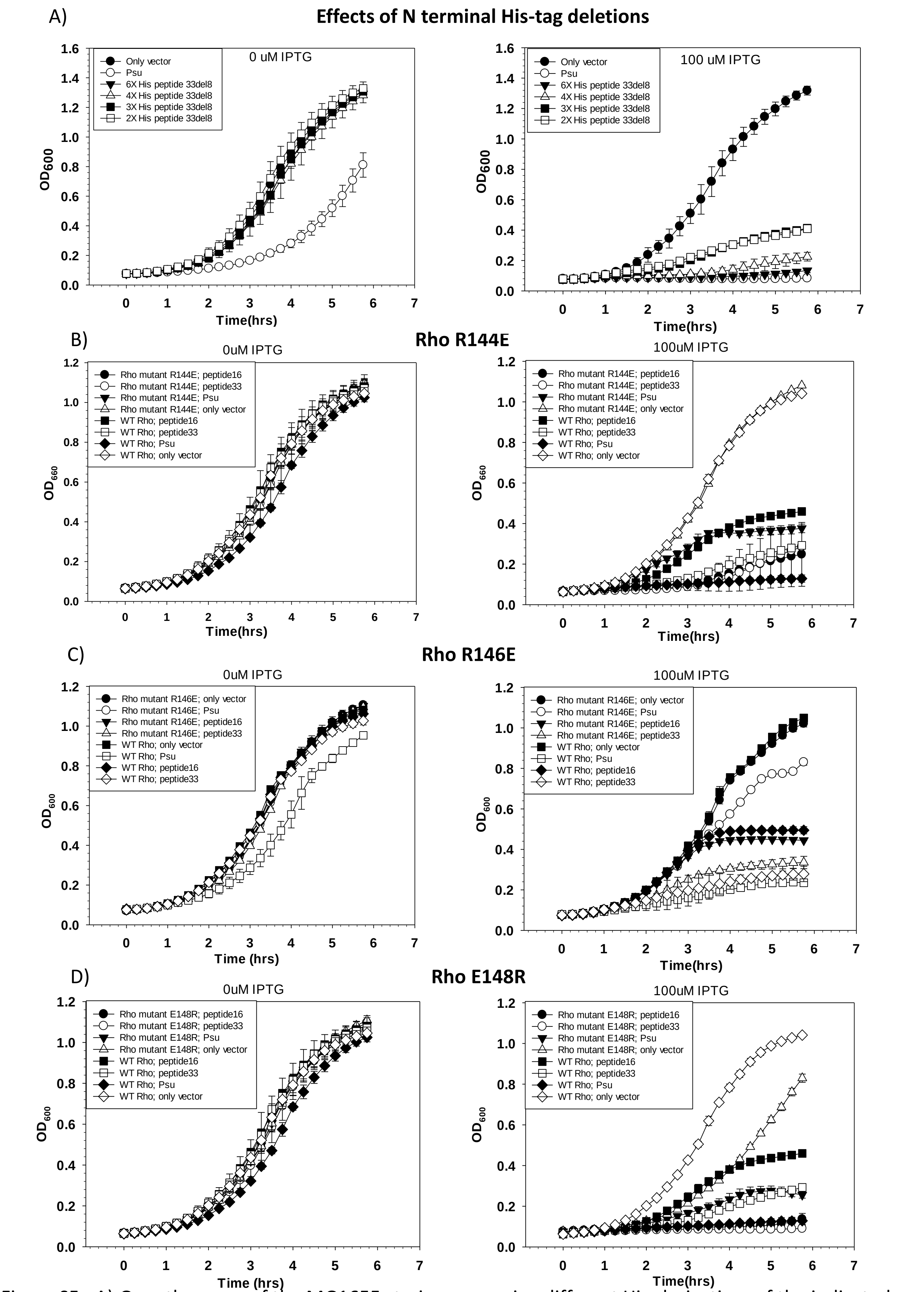

Figure S5: A) Growth curves of the MG1655 strains expressing different His-derivatives of the indicated peptides. B-D) Growth curves MG1655 strain having different Rho mutants upon expressions of Psu and peptides under different conditions. Experiments were done in the same way as in figure 2A. Standard deviations were obtained from 3 independent measurements.

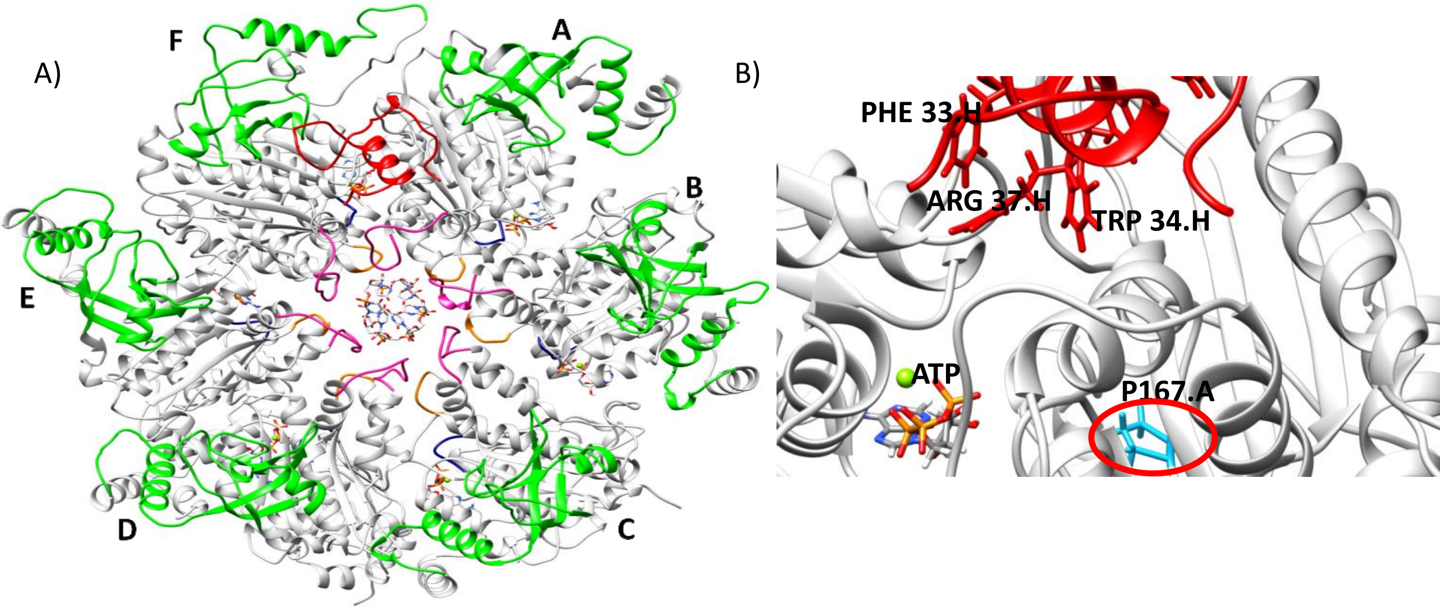

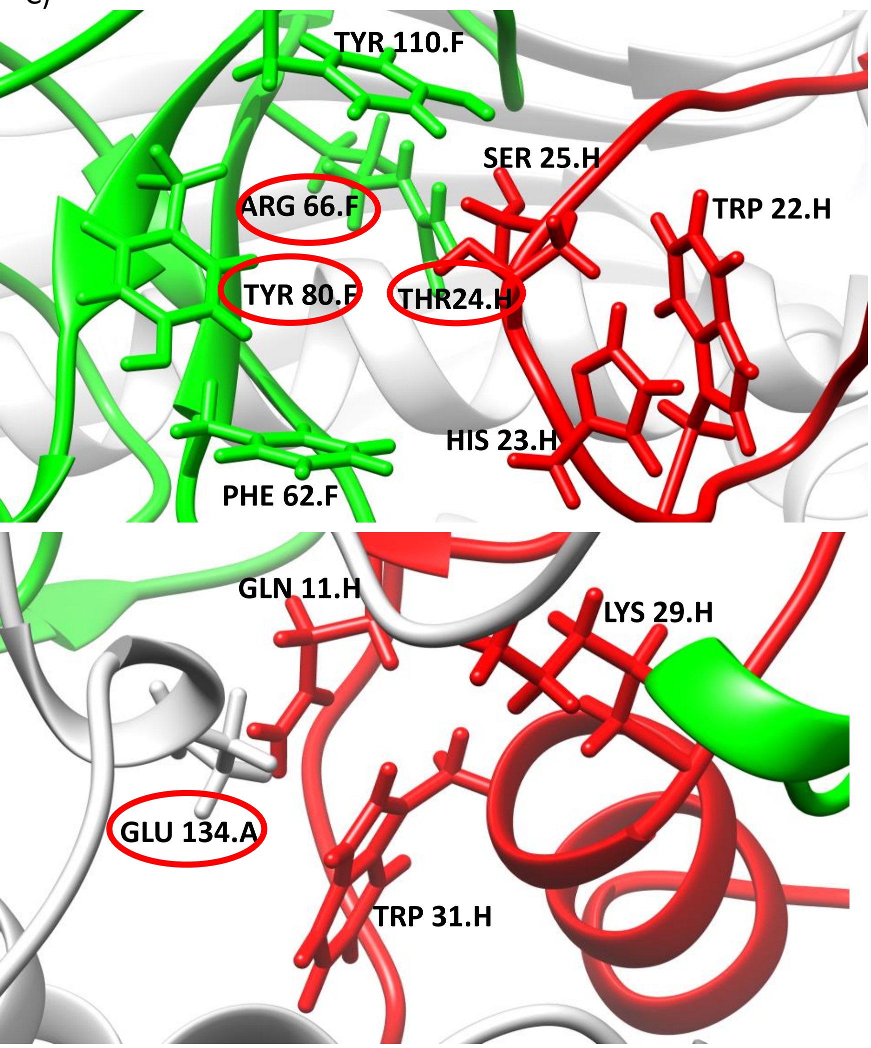

D)

Peptide33	Rho	Distance (Å)
THR 8	ARG 92.A	4.6
	LEU 132.A	5.9
	LEU 17.F	5.5
ALA 10	ARG 92.A	5/0
	LEU 132.A	5.9
	LEU 17.F	4.9
GLN 11	ARG 92.A	5.1
	LEU 132.A	5.5
	**GLU 134.A**	5.7
	GLU 248.A	5.8
	ARG 252.A	5.6
	LEU 17.F	5.3
TRP 22	PHE 62.F	6.0
	**TYR 80.F**	9.5
	TYR 110.F	5.3
HIS 23	PHE 62.F	5.7
	**TYR80F**	8.3
THR 24	PHE 62.F	5.6
	**TYR80 F**	5.4
	TYR 110.F	5.6
SER 25	ARG66.F	4.6
	**TYR 80.F**	7.7
	TYR 110.F	4.3
LYS 29	**GLU 134.A**	5.6
TRP 31	**GLU 134.A**	4.5
	PRO167.A	25.4
PHE 33	ASN292.A	5.4
TRP 34	ASN292.A	5.1
	ARG 296.A	5.5
ARG 37	ASN292.A	5.2
	ARG 296.A	5.8

Figure S6: Docking of peptide 33 on the Rho hexamer and the key residues at the peptide-Rho interface: (**A**) The cartoon representation of Rho hexamer-peptide33 complex. Peptide33 (red) was modelled onto the PBS region of the Rho hexamer (in green) in between the latter's A and F chains. **B**) Location of P167 on Rho. (**C**) The interface residues that come within the interacting distances from both the Rho and peptide 33 are shown. The three key residues R66 (F chain), Y80 (F chain) and E134 (A chain) are encircled as point mutations in these three positions were used for experimental verifications. (**D**) Pairwise distances (Å) between the interface residues of Rho and peptide33 are listed. The Rho P167 position is highlighted.

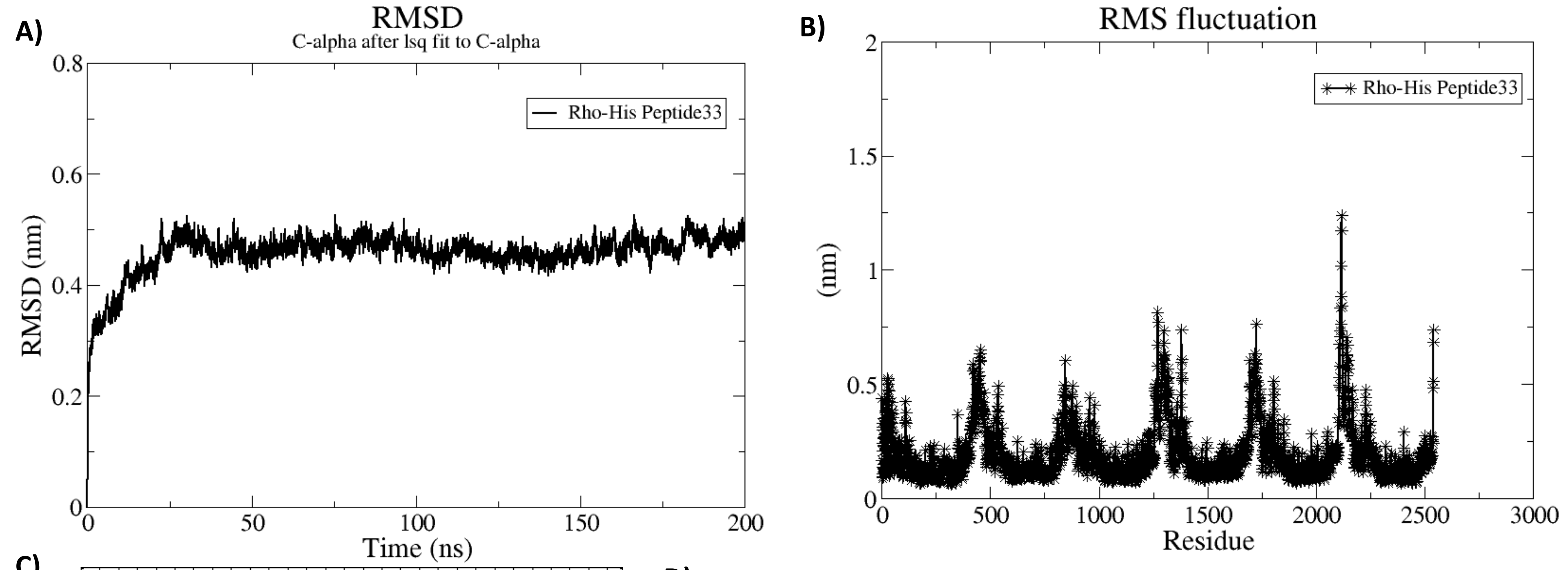

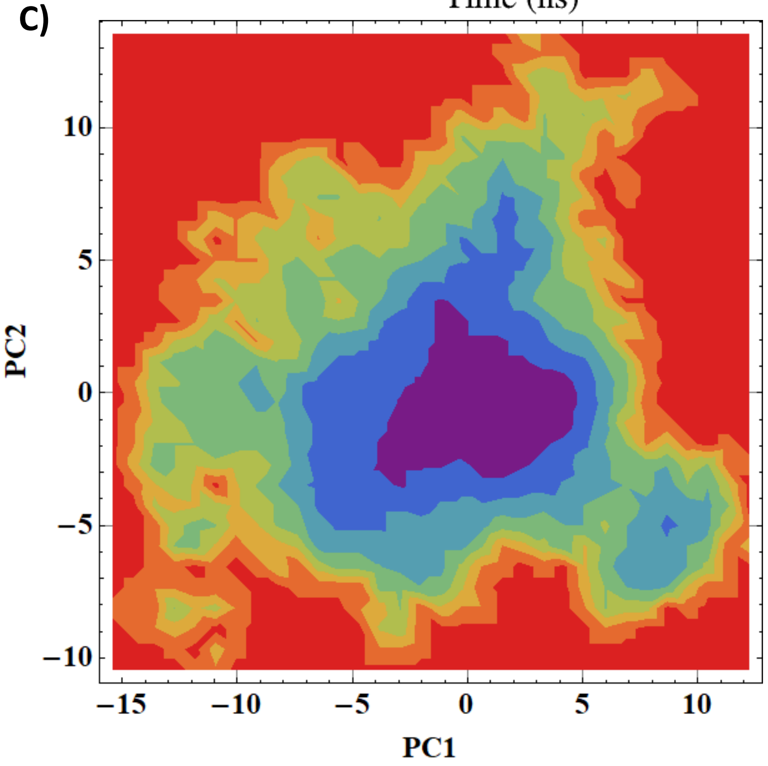

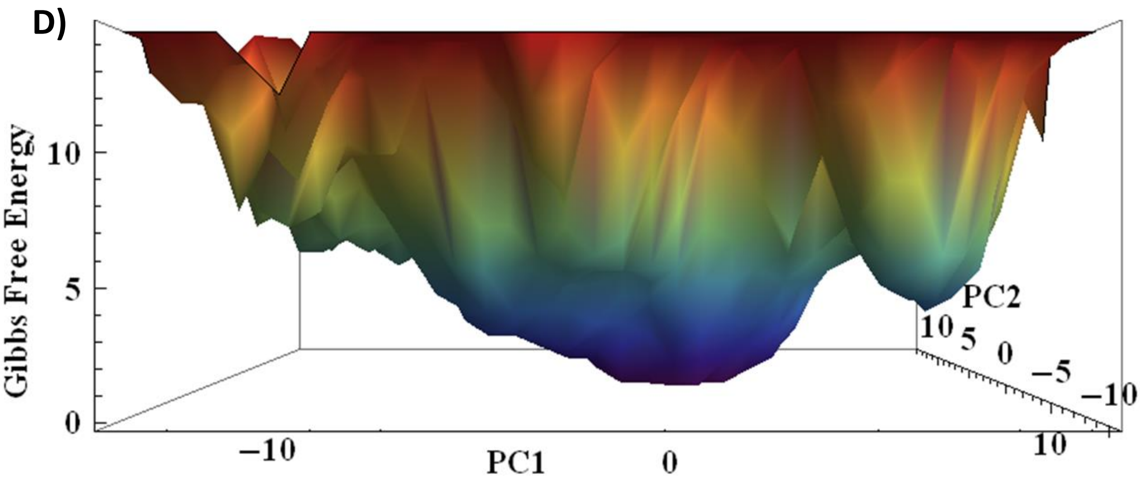

Figure S7: MD simulation of Rho – Peptide33 complex. (A) The RMSD (C-alpha atoms) of the complex raised to 0.5 nm till 30ns, and then it remained stable for the rest of the simulation period of 200 ns. **(B)** The RMS fluctuations of the interface residues in the complex for 200ns time scale indicate that the complex is stable. **(C)** and **(D)** the energy contour plots obtained from the FEL analysis. Single energy minimum cluster can be seen in 2D and 3D FEL plots, from which the representative complex structure was retrieved.

Figure S7

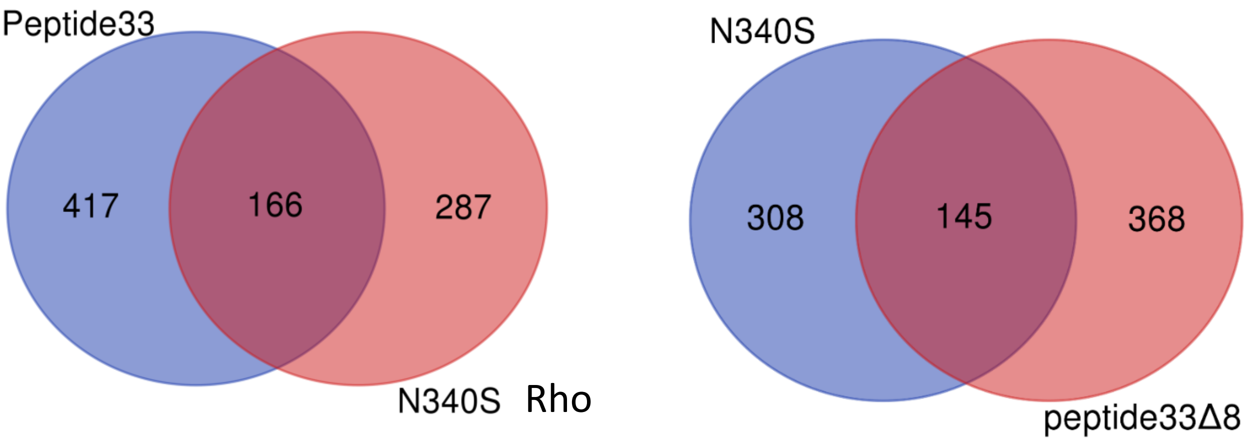

Figure S8: Overlapping circles showing the common genes that are upregulated in the presence of Rho mutant N340S and upon expressions of the peptides obtained from the microarray profiles.

Table S1: Mean C_t values for each biological replicates (mean values obtained from two technical replicates).

(rpoC is the reference gene)

Test genes/ terminators	**Peptide/Psu**	**Biological rep 1**		**Biological rep2**		**Biological rep 3**		**Biological rep 4**		**Biological rep 5**	
		rpoC	***test gene***	***rpoC***	***test gene***	***rpoC***	***test gene***	***rpoC***	***test gene***	***rpoC***	***test gene***
trpt'	**Only vector**	23.0896994	25.5625322	21.5495065	28.1	20.5651927	27.5461337				
	**Psu**	21.0280221	21.3656251	20.3254651	24.8293295	21.6134747	25.1765066				
	**Peptide16**	21.5026036	22.9576662	22.6926838	28.3419723	20.8680711	26.8168238				
	**Peptide33**	22.7444693	21.5527475	22.4231359	27.0658006	21.6836842	26.3274123				
trac	**Only vector**	21.0567043	29.7450371	18.5271305	28.9092438	21.3965286	28.7744063				
	**Psu**	20.218344	25.1187053	19.919429	25.2231549	21.9085837	24.0903747				
	**Peptide16**	21.7950316	28.2512113	19.4871482	25.6272822	22.1495386	25.8344289				
	**Peptide33**	21.465511	27.2453379	19.0835514	27.202756	22.1217937	26.5764855				
yafW	**Only vector**	20.6948547	26.3705025	19.0076298	26.3228124	20.0863095	28.3722121				
	**Psu**	19.7599354	22.0015106	20.91785	23.9169055	20.7462307	25.8142684				
	**Peptide33**	20.7375078	26.885869	20.1110664	25.224911	20.8703413	26.6348747				
	**Peptide33Δ8**	20.0897398	25.339613	20.5705121	26.8743751	20.9787186	27.7945673				
ydeT	**Only vector**	20.6948547	25.6538315	19.0076298	27.2349733	22.5065648	31.4432815				
	**Psu**	19.7599354	23.04638	20.91785	25.8309424	21.195	28.45191				
	**Peptide33**	20.7375078	27.2334738	20.1110664	24.7701738	22.0147812	29.4258746				
	**Peptide33Δ8**	20.0897398	25.0229349	20.5705121	27.0548558	22.2085836	29.5754867				
yeeE	**Only vector**	19.9582948	24.5504402	18.1316134	28.9237228	22.247659	31.5694831	21.0044333	30.56792206	20.08630953	30.97205
	**Psu**	19.10	26.2990538	19.44	25.3291889	20.41	27.120429	19.7923146	26.27691624	20.74623069	27.14496
	**Peptide33**	20.36	29.5253737	18.77	25.7695861	20.97	29.5014779	21.6160609	32.95696427	20.87034128	27.26781
	**Peptide33Δ8**	19.49	30.271415	19.12	29.269764	20.90	28.0431534	20.3989247	28.13900393	20.97871857	29.39737
yegQ	**Only vector**	21.0044333	27.1365837	22.247659	27.7604568	18.1316134	23.4858852	19.9582948	26.10558029		
	**Psu**	19.7923146	20.876753	20.4082479	23.3049032	19.4406334	22.0677349	19.1008158	21.82962405		
	**Peptide33**	21.6160609	28.2374271	20.9674999	27.0527386	18.7689655	22.1192272	20.3550176	26.07776448		
	**Peptide33Δ8**	20.3989247	24.986337	20.9042323	25.2155715	19.1193183	24.1121971	19.48885	24.66639227		

fimD	**Only vector**	23.1772031	30.9435318	21.9813008	30.9435318				
	**Psu**	21.3540865	28.1327497	20.5151124	26.3289041				
	**Peptide33**	21.7890561	28.8726279	20.0114613	26.5638377				
	**Peptide33Δ8**	21.7372703	28.1113948	21.2217724	28.5139071				
ykfG	**Only vector**	23.1772031	28.5811132	21.9813008	28.4896077	20.0863095	27.9293434		
	**Psu**	21.3540865	24.0837911	20.5151124	20.8232278	20.7462307	25.09788		
	**Peptide33**	21.7890561	27.6145359	20.0114613	26.3694668	20.8703413	26.29277		
	**Peptide33Δ8**	21.7372703	26.7540561	21.2217724	26.7210274	20.9787186	26.8180041		
gfcB	**Only vector**	17.5385444	28.0568258	19.852364	29.7786878	17.5385444	21.1715492		
	**Psu**	18.9810324	28.2860335	19.0551363	25.4224964	18.9810324	22.2406077		
	**Peptide33**	18.6941118	28.7830237	19.0653051	28.4394601	18.6941118	20.6944356		
	**Peptide33Δ8**	19.1616865	27.5788924	19.2551407	28.7598896	19.1616865	20.7811444		
thiM	**Only vector**	19.0076298	27.9903258	20.6948547	27.5794764	21.2999785	27.9653071		
	**Psu**	20.91785	28.4471115	19.7599354	27.5794764	20.8979257	24.7839779		
	**Peptide33**	20.1110664	26.7346694	20.7375078	27.1850843	20.1428801	25.6973881		
	**Peptide33Δ8**	20.5705121	28.1775807	20.7375078	26.7077265	20.6897259	26.2336074		
mgtA	**Only vector**	21.6778423	26.2260747	22.0283556	25.6508971	22.3518454	26.7299551		
	**Psu**	21.9816222	23.1486691	21.404199	22.2214482	21.812833	23.458671		
	**Peptide33**	21.1386201	22.9644923	20.8524515	22.6621073	20.5299796	22.5100233		
	**Peptide33Δ8**	21.2365148	24.117358	22.316973	23.9821035	20.5108365	23.0957868		
ykfH	**Only vector**	22.0283556	27.0586422	22.3518454	27.9196757	22.5500193	28.0658207	23.4454174	28.83504559
	**Psu**	21.404199	19.4677882	21.812833	20.1515144	22.6983784	21.1124307	22.7579155	21.11529747
	**Peptide33**	20.8524515	24.1854593	20.5299796	24.3255354	22.7703775	26.5333249	21.701164	25.45538747
	**Peptide33Δ8**	21.316973	27.7467685	21.5108365	26.9529227	21.9857677	27.7620713	21.7011053	27.38670108
setC	**Only vector**	22.0283556	32.4485406	22.3518454	32.4663151				
	**Psu**	21.404199	25.7241658	21.812833	26.6784447				
	**Peptide33**	20.8524515	29.2911484	20.5299796	29.0166323				
	**Peptide33Δ8**	21.316973	31.9282776	20.5108365	31.6704435				

Table S2: Oligonucleotides used in this study.

Oligos	Sequence	Description
RS83	ATAAACTGCCAGGAATTGGGGATC	5'-biotinylated RS58
RS177	TTGTGAGCGCTCACAATTCGGATATA TATTAACAATTACCTG	Reverse oligonucleotide with *lac* operator sequence, used to generate roadblock downstream of *rut* sites of T7A1-*trp t'* template
RS952	TGACAATTAATCATC GGCTCGTATAATGT	P_{tac} Promoter sequence, before MCS (for sequencing)
RS1230	AGT TCGGCATGGGGT CAG GTGGGACC	pNL150 vector specific Reverse Primer, 100bp downstream of gene
RS1705	GCGCAAGCTTTTATAC ACT GAC TGA CGT ATG CCA GTT GCG C	Reverse primer with Hind-III site for cloning 33-peptide (histag/peptide/stop) w/o vector sequence in pNL150
RS1706	GCGCAAGCTTTTATACACTGACTGAC GTGATGCCAGTGCGC	reverse primer Hind-III site for cloning 16-peptide (histag/peptide/stop) w/o vector sequence in pNL150
RS1721	CCGCGAATTCATGCATCATCATCATC ATCATACCCCTGCGCAGTTCATATTT TTTCGCG	forward primer with EcoRI site for cloning His-peptide sequence for 33 in pNL150
RS1722	CCGCGAATTCATGCATCATCATCATC ATCATACCCCTGCGCAGGGCATGATT TTTTCGG	forward primer with EcoRI site for cloning His-peptide sequence for 16 in pNL150
RS1725	GCGCAAGCTTTTAATCTGTATCAGGC TGAAAATCTTCTCTCATCCGCC	RP with HindIII site for cloning 33 peptide in pSTKT
RS1745	GCGC AAG CTT TCA CTG ACT GAC GTG ATG CCA GTT GCG C	RP with HindIII site for cloning His-WT peptide alpha7 with adjacent vector sequence in pnl150
RS1795	GCGCGAATTCATGACCCCTGCGCAG	FP with EcoRI site for cloning Non-His 33 peptide along with vector sequence
RS1814	TAAATCAGAACGCAGAAGCGG	FP for peptide CTD deletion by SDM
RS1815	CTGAAAATCTTCTCTCATCCGCC	RP for Δ4 CTD peptide deletion by SDM
RS1816	ATCTTCTCTCATCCGCCAAAACAGCC	RP for Δ6 CTD peptide deletion by SDM
RS1817	TCTCATCCGCCAAAACAGCCAAGC	RP for Δ8 CTD peptide deletion by SDM
RS1880	GCGCGAATTCATGCATCATCATCATA CCCCTGCGCAGTTCATATTTTTTCGC GTAAAAGTGCGC	FP for 4X N-ter his cloning of 33 peptide
RS1881	GCGCGAATTCATGCATCATCATACCC CTGCGCAGTTCATATTTTTTCGCGTA AAAGTGCGC	FP for 3X N-ter his cloning of 33 peptide
RS1882	GCGCGAATTCATGCATCATACCCCTG CGCAGTTCATATTTTTTCGCGTAAAA GTGCGC	FP for 2X N-ter his cloning of 33 peptide
RS1977	GCGCAAGCTTTTACCGCCAAAACAG CCAAGCTTTTACACTGACTGACG	Peptide 33Δ10 CTD RP
RS1978	GCGCAAGCTTTTAAAACAGCCAAGC TTTTACACTGACTGACG	Peptide 33Δ12 CTD RP
